# The Influence of Plasticizers and Accelerated Ageing on Biodegradation of PLA under Controlled Composting Conditions

**DOI:** 10.3390/polym15010140

**Published:** 2022-12-28

**Authors:** Pavel Brdlík, Jan Novák, Martin Borůvka, Luboš Běhálek, Petr Lenfeld

**Affiliations:** Faculty of Mechanical Engineering, Technical University of Liberec, Studentska 1402/2, 46117 Liberec, Czech Republic

**Keywords:** PLA films, plasticizers, biodegradation, ageing, composting

## Abstract

The overall performance of plasticizers on common mechanical and physical properties, as well as on the processability of polylactic acid (PLA) films, is well-explored. However, the influence of plasticizers on biodegradation is still in its infancy. In this study, the influence of natural-based dicarboxylic acid-based ester plasticizers (MC2178 and MC2192), acetyl tributyl citrate (ATBC Citroflex A4), and polyethylene glycol (PEG 400) on the biodegradation of extruded PLA films was evaluated. Furthermore, the influence of accelerated ageing on the performance properties and biodegradation of films was further investigated. The biodegradation of films was determined under controlled thermophilic composting conditions (ISO 14855-1). Apart from respirometry, an evaluation of the degree of disintegration, differential scanning calorimetry (DSC), thermogravimetric analysis (TGA), Fourier transform infrared spectroscopy (FT-IR), and scanning electron microscopy (SEM) of film surfaces was conducted. The influence of melt-processing with plasticizers has a significant effect on structural changes. Especially, the degree of crystallinity has been found to be a major factor which affects the biodegradation rate. The lowest biodegradation rates have been evaluated for films plasticized with PEG 400. These lower molecular weight plasticizers enhanced the crystallinity degrees of the PLA phase due to an increase in chain mobility. On the contrary, the highest biodegradation rate was found for films plasticized with MC2192, which has a higher molecular weight and evoked minimal structural changes of the PLA. From the evaluated results, it could also be stated that migration of plasticizers, physical ageing, and chain scission of films prompted by ageing significantly influenced both the mechanical and thermal properties, as well as the biodegradation rate. Therefore, the ageing of parts has to be taken into consideration for the proper evolution of the biodegradation of plasticized PLA and their applications.

## 1. Introduction

In 2020, more than 29 million tons of plastic post-consumer waste were collected in the EU27 + 3 [1]. Although compared to previous years, the ratio of recycling and energy recovery is increasing, still, more than 23% of plastic waste ends up in landfills [1]. If the world population and demand for plastics continues within the current trend, the plastics demand will achieve 1000 million tons by 2050 [2]. Therefore, it is evident that waste management and environmental pollution will continue to increase. Undoubtedly, the largest end-used market is the packing industry (40.5%), where the most used materials are polyethylene terephthalate (PET), polyethylene (PE), and polypropylene (PP). Despite being potentially recyclable, monomaterial flexible packaging solutions based on these petroleum-based polymers, in most cases, end up in energy recovery or landfills. The environmentally friendly solution could be the incorporation of biodegradable polymers that are made from renewable sources. Nevertheless, the amount of biodegradable biopolymers in this segment is still lower than 1% [1,3].

There are currently several commercially available biodegradable biopolymers on the market, such as polylactic acid (PLA), polyhydroxyalkanoates (PHA), polybutylene adipate terephthalate (PBAT), and starch-based blends. These materials represent nearly 60% of thermoplastic biopolymer production capacities [3]. Especially, PLA, due to its high transparency, gloss, scratch resistance, and relatively high strength and toughness, could be an interesting alternative to petroleum-based polymers in the packing industry and in agriculture and textile segments. However, PLA is further characterized by low ductility and resistance to fracture. In order to improve the utility properties of PLA, plasticizers are often added to the PLA. Plasticizers, most-often low molecular weight polymers or oligomers, evoke the enhancement of macromolecular distance that ensures that the intermolecular forces decrease and increase the mobility of the system. Consequently, the glass transition temperature is shifted to lower temperatures and brittleness is reduced. Phthalic acid has become the most-used class of plasticizers in the 21st century [4]. However, issues such as problematic degradation, migration, and negative impact on human health were a catalyst for the development of new non-toxic, environmentally friendly, and biodegradable green-plasticizers [4,5,6]. Plenty of researchers have focused on this issue in the last two decades. Emad et al. [7] evaluated the thermal stability, and mechanical and morphological properties of PLA plasticized with epoxidized palm oil. A remarkable increase in the ductility of PLA by the addition of epoxidized cottonseed oil (ESCO) was presented in the study of Verdi et al. [8]. Ljungberg et al. [9] presented a decrease in the storage modulus and thermal properties (melting and glass transition temperatures) of PLA plasticized with tributyl citrate (TBC). Maiza et al. [10] studied the plasticizing effect of triethyl citrate (TEC) and acetyl tributyl citrate (ATBC). Besides a decrease in the glass transition temperature, dynamic storage modulus, and thermal stability, no color changes in the PLA films were observed in this study. Special effort was given to analyzing the applicability of poly(ethylene glycol) (PEG) plasticizer in the past few decades [11,12,13]. Courgneau et al. [14] compared the mechanical and thermal properties of PLA plasticized with ATBC and PEG in the content range from 2.5 wt. % to 20 wt. %. The improvement of mechanical properties is not the only aspect that has to be taken into consideration for the evaluation applicability of plasticizers in the packaging segment. Other fundamental aspects are film barrier properties (oxygen and water permeability) and migration of plasticizers. Courgneau et al. [14] presented that ATBC plasticizer maintains its gas barrier properties and water vapour transmission up to 13 wt. %. However, the barrier properties decrease in PLA already at 9 wt. % content of PEG plasticizer. On the contrary, Sessini et al. [15] achieved increasing hydrophobicity by incorporating 10 wt. % of limonene oxide (LO) produced from the peel of citrus fruits. Another issue is the migration of plasticizers that results in changes in physical and mechanical properties and color changes in plasticized PLA [10]. Kodal et al. [16] declared, through investigation with scanning electron microscopy, that the migration of PEG 400 in the amount of 20 wt. % content occurred. Also, Tsou et al. [17] reported some migration tendencies of ATBC. On the contrary, in the study of Burgos et al. [18], changes in thermal, mechanical, structural, and barrier properties that could be linked to the migration of oligomeric lactic acid (OLA) plasticizer even at 25 wt. % have not been observed.

Regarding the previous summary, the influence of chemistry, polarity, content, and molecular weight of plasticizers on thermal, mechanical, and barrier properties, as well as migration, has been discussed many times. However, from the view of environmental aspects, one of the most important questions, “How the plasticizers influence biodegradation of PLA,” is still not well explained. PLA is polyester, where hydrolysis and enzymatic/microbial activity are the dominant degradation mechanisms. The biodegradation process could, despite the influence of environmental characteristics such as pH, temperature, moisture, and atmosphere composition, occur from several weeks up to years. For the degradation of PLA, temperature is one of the most critical parameters. Itävaara et al. [19] and Kale et al. [20] reported that faster biodegradation of PLA is achieved in thermophilic conditions compared to mesophilic ones. The higher temperature of the medium evokes a decrease in intermolecular binding forces that cause a more straightforward hydrolysis reaction and the attachment of microbes/enzymes. Therefore, incorporating low molecular weight plasticizers into PLA could evoke faster biodegradation rates due to the increased macromolecular chain mobility. On the contrary, the increasing macromolecule chain mobility could stimulate changes in morphology structure and enhance the crystallinity degree. Kolstad et al. [21] reported very poor biodegradability of semicrystalline PLA compared to amorphous PLA. It has been reported that the amorphous regions are easily assimilated by microorganisms [22]. Consequently, the biodegradation rate of PLA could also decrease with the incorporation of plasticizers. Furthermore, the polar character and end-chain group of plasticizers influence oxygen permeability and hydrophobicity. Another critical factor which affects biodegradation is the chemical composition of plasticizers. Regarding the previous summary, it is obvious that the effect of plasticizers on biodegradation rate complicates many factors and cannot be simply predicted. Therefore, the current work is dedicated to investigating the influence of the most often used plasticizers, PEG and ATBC, on the biodegradation rate of PLA under controlled thermophilic composting (ISO 1455-1). Besides PEG and ATBC, new plasticizers, MC 2178 and MC2192, based on 100% biobased dicarboxylic acid esters, were investigated. The reason behind this was to study the determining influence of the molecular weight of plasticizers on the biodegradation rate. The influence of ageing on the utility properties of plasticized PLA is another underexplored area of this research environment. Therefore, the influence of accelerated ageing on the changes in mechanical and thermal properties, and also on biodegradation, was also investigated.

## 2. Materials and Methods

The commercial 100% biobased PLA Luminy L 130 was purchased from Total Energies Caorbion (Gorinchem, The Netherlands). Luminy L 130 is high heat (the melting point is 175 °C, glass transition temperature is 60 °C), medium flow PLA (melt flow index 23 g/10 min, ISO 1133-A) homopolymer with minimal 99% L-isomer stereochemical purity. PLA Luminy L 130 was plasticized with PEG 400 (Sigma-Aldrich, Taufkirchen, Germany), ATBC Citroflex A4 (Vertellus Holding LLC, Linz, Austria), dicarboxylic acid-based plasticizers MC 2178 and MC 2192 (Emery Oleochemicals GmbH, Dusseldorf, Germany). The characteristic properties of plasticizers are listed in Table 1. All the plasticizers have food contact approval.

### 2.1. Preparation of PLA Films

Before the production process, any moisture from PLA pellets was removed using the vacuum oven VD53 (Binder, Germany) at 80 °C for 12 h. Further, the PLA was plasticized in compounder Collin ZK 25 P (COLLIN Lab & Pilot Solutions GmbH, Maitenbeth, Germany) equipped with automatic volumetric DVL LIQUIDOSER (Moretto, Italy), which is designed especially for dosing liquid additives. The plasticizers are commonly used in the range from 5 to 20 wt. %. The lower concentration of plasticizer ensures a lower potential of migration and better miscibility with PLA. However, the improvement in flexibility and fracture resistance is lower. The influence on barrier properties will be lower than in higher concentrations. Consequently, a lower influence of plasticizer on biodegradability of PLA could be assumed. Therefore, the dosing of plasticizers to compounder was established to achieve a final concentration of 15 wt. % in PLA. The temperature profile from 145 °C to 165 °C and speed of 160 rpm was used for PLA compounding. The plasticized PLA was further transformed in an extrusion head set up to 180 °C and pelletizer running at 3000 rpm. Before film processing, another vacuum drying process for 12 h at 80 °C was incorporated for moisture removing. The PLA films were extruded on twin screw extruder MC 15 HT (Xplore, Netherlands) and equipped with flat film die (0.4 mm gap size) at the constant melt temperature of 185 °C and 100 rpm screw speed.

### 2.2. Accelerated Ageing

The ageing process was performed in climatic chamber SUN 3600 (Weiss Technik, Balingen, Germany) incorporated with two 4 kW metal halide (MH) lamps. The climatic chamber made it possible to use irradiation intensity from 400 to 1150 W/m^2^ in the spectral region of radiation from 300 nm to 2450 nm. Consequently, simulation of solar radiation at ground level that is defined in standard IEC 60068-2-5 could be tested. The condition of ageing was used with respect to DIN 75 220 standard. After the conditioning process (25 °C, 50% relative humidity, 240 h) in the climatic chamber (Teseco, Kostelec nad Orlicí, Czech Republic), films were exposed to constant radiation intensity 1000 W/m^2^ for 240 h. The recommended temperature of chamber 42 °C was due to the potential decrease in glass transition in plasticized PLA films decreased to 28 °C. The relative humidity was adjusted to 65%, an average value in the summer months in the Czech Republic [30].

### 2.3. Mechanical Properties

The influence of plasticizers and accelerated ageing process on mechanical properties were evaluated by determination of tensile modulus (E_t_), tensile strength (σ_m_), and nominal tensile strain at break (ε_tb_). TiraTest tensile testing machine (Tira GmbH, Schalkau, Germany), which is equipped with KAF type of load cells (ranging from 0 to 1000 N, sensitivity 2.0 mV/V) and with WA-type series displacement transducers (max. linearity deviation: − 0.13% and nominal supply voltage: 80 mV/V), was used for this study. The PLA films were, according to ISO 527-3 standard, trimmed to a size of 15 ± 0.2 mm width and 160 ± 2 mm length. Before testing, samples were conditioned in the climatic chamber (Teseco, Kostelec nad Orlicí, Czech Republic) at temperature of 25 °C and 50% relative humidity for 240 h. According to the ISO 527 standard, load speed of 1 mm/min was used for determination of tensile modulus. For the tensile strength and strain at break, load speed of 5 mm/min was applied. The results were evaluated from 15 measurements.

### 2.4. Rheological Properties

The evaluation of rheological properties of conditioned samples was further incorporated for determination of accelerated ageing impact on the stability of plasticized PLA films. The changes in rheological properties were evaluated with melt flow rate (MVR) analysis according to ISO 1133-1 (190 °C/2.16 kg). The Ceast 7028 (Instron, Buckinghamshire, UK) melt flow tester from company Instron (Buckinghamshire, UK) was used.

### 2.5. Analysis of Biodegradability under Thermophilic Composting

The method adapted from the ISO 14855-1 standard was used to analyse the influence of plasticizers on the biodegradation kinetics of PLA films in compost thermophilic (58 °C) environment. This method is based on evaluation of carbon dioxide amount evolution during the microbial degradation. The released carbon dioxide was detected with spirometer ECHO (ECHO d.o.o., Slovenske Konjce, Slovenia). Regarding the standard, the 10 g films were trimmed to pieces sized about 1 cm × 1 cm and placed into 2.8 l cylindrical hermetic vessels that contained 150 g of compost. The commonly available compost from the company AGRO CS (Říkov, Czech Republic), with 5.2 pH (measured by the Volcraft PH-100ATC pH meter, Conrad Electronic s.r.o., Praha, Czech Republic ), was used in this study. Before the biodegradation experiment, 50% humidity content of compost was adjusted by the halogen moisture analyser Mettler Toledo™ HX204 (Mettler Toledo, Columbus, OH, USA) and pebbles or other foreign objects larger than 2 mm were removed from the compost. For the proper microbial activity control of the compost, vessels with microcrystalline cellulose (Sigma-Aldrich, Saint-Quentin-Falavier, France) were used. The vessels were shielded from the light. The vessels were opened once a week and the compost was stirred to ensure an even distribution of moisture. Each biodegradation analysis was performed in duplicity. The percentage of biodegradation was determined in accordance with the following equation:

(1) $$D_{t}=\frac{{\left({\mathrm{CO}}_{2}\right)}_{T}-{\left({\mathrm{CO}}_{2}\right)}_{B}}{T_{{\mathrm{hCO}}_{2}}}\cdot 100$$

where (CO_2_)_T_ is the cumulative amount of carbon dioxide evolved in the composting vessel containing the test material, (CO_2_)_B_ is the mean cumulative amount of carbon dioxide evolved in the blank vessels, and (T_hCO_2__) is the theoretical amount of carbon dioxide that can be produced by the test material (all in g/vessel).

The theoretical amount of carbon dioxide can be determined via the following equation:

(2) $$T_{{\mathrm{hCO}}_{2}}=M_{\mathrm{TOT}}\cdot C_{\mathrm{TOT}}\cdot \frac{44}{12}$$

where M_TOT_ is the total number of dry solids in the test material introduced into the composting vessel at the start of the test (in g), C_TOT_ is the proportion of total organic carbon in the total dry solids in the test material (in g/g), and 44 and 12 are the molecular mass of carbon dioxide and the atomic mass of carbon, respectively. The individual proportions of total organic carbon of PLA films are listed in Table 2.

With respect to changes in organic carbon content, there is no possible way to do any supplemental analysis during a spirometry test. Therefore, the parallel experiment was carried out. The plasticized PLA films (size of 100 mm × 40 mm) were exposed to the same compost at the same condition as was used in above-introduced thermophilic composting analysis. The films were subjected to evaluation of disintegration degree every 14 days, being removed and conditioned (25 °C, 240 h, 50% relatively humidity) in climatic chamber (Teseco, Czech Republic). However, after 28 days, the experiment was finished because of intensive disintegration of some plasticized PLA films. The structural, thermal, and chemical changes, as well as surface roughness, were evaluated with differential scanning calorimetry (DSC), furrier transform infrared spectroscopy (FT-IR), thermogravimetric analysis (TGA), and scanning electron microscopy (SEM). The results were evaluated from 3 measurements. Therefore, the standard deviation has not been specified, only average values were provided.

### 2.6. Differential Scanning Calorimetry (DSC)

Thermal properties and structural changes were evaluated in a calorimeter DSC 1/700 (Mettler Toledo, Greifensee, Switzerland). Samples of approximately 5 mg taken from cross-section of the PLA films were sealed in aluminium pan and placed into DSC chamber where the constant nitrogen flow of 50 mL/min was adjusted. The samples were heated in temperature profile from 0 °C to 200 °C with heating rate of 10 °C/min. The samples were kept isothermal for 180 s at 200 °C, then the cooling process at rate of 10 °C/min was initiated. The glass transition temperature (T_g_), cold crystallisation temperatures and enthalpies (T_cc_, ∆H_cc_), melting temperatures and enthalpies (T_m_, ∆H_m_), and primary crystallisation temperatures and enthalpies (T_c_, ∆H_c_) were evaluated. The degree of crystallinity (X_c_) was determined through the following equation:

(3) $$X_{C}=\frac{\Delta H_{m}-\Delta H_{c}-\Delta H_{\mathrm{cc}}}{\Delta H_{m}^{0}\Delta w_{m}}=\frac{\Delta H_{}}{\Delta H_{m}^{0}\Delta w_{m}}\cdot 100$$

where ∆H^0^_m_ is the melting enthalpy of 100% crystalline PLA (106 J/g), w_m_ is the mass fraction of PLA in the composites, and the ∆H is the enthalpy balance.

### 2.7. Fourier Transform Infrared Spectroscopy (FT-IR)

The chemical changes in the PLA films were analysed using an infrared spectrometer Nicolet iS10 (Thermo Scientific, Waltham, MA, USA) in Attenuated Total Reflectance (ATR) mode using diamond crystal. The FTIR-ATR spectra were recorded in the range of 400–4000 cm^−1^ by averaging 64 scans and using a resolution of 2 cm^−1^.

### 2.8. Thermogravimetric Analysis (TGA)

Thermal stability was evaluated using TGA2 instrument (Mettler Toledo, Switzerland). The samples were prepared with the same principle as for DSC analysis. They were taken from the cross-section of films in weights of 5 ± 0.5 mg. Further, heating from 50 °C to 600 °C at the heating ramp of 10 °C/min in nitrogen atmosphere was performed and the decomposition temperature at 5% weight loss (T_5%_) and 50% weight loss (T_50%_) were evaluated. 

### 2.9. Scanning Electron Microscopy (SEM) Analysis

The mechanism of degradation (surface, bulk) and surface changes were observed with field emission scanning electron microscopy (FE-SEM). To this purpose, the microscope TESCAN MIRA 3 (Tescan, Brno, Czech Republic) instrument with an accelerated voltage of 3 kV was used. The test samples were, prior to analysis, coated with 1 nm of platinum using Q150R ES (Quorum Technologies, UK).

## 3. Results

### 3.1. Mechanical Properties

**Figure 1:**
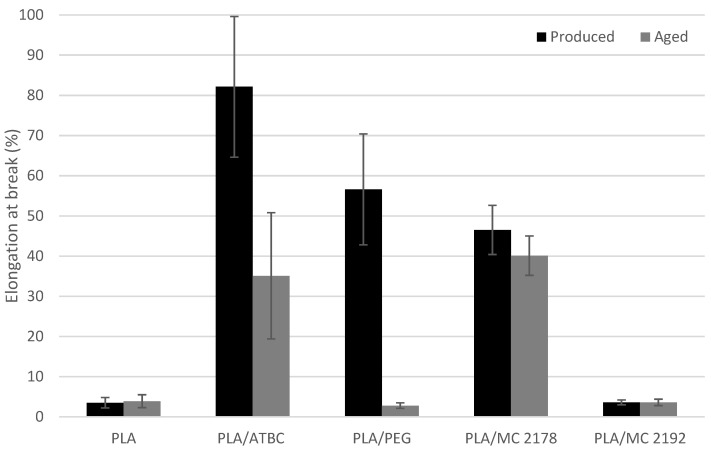
The evaluated results of elongation at break for as-produced and aged PLA films.

**Figure 2:**
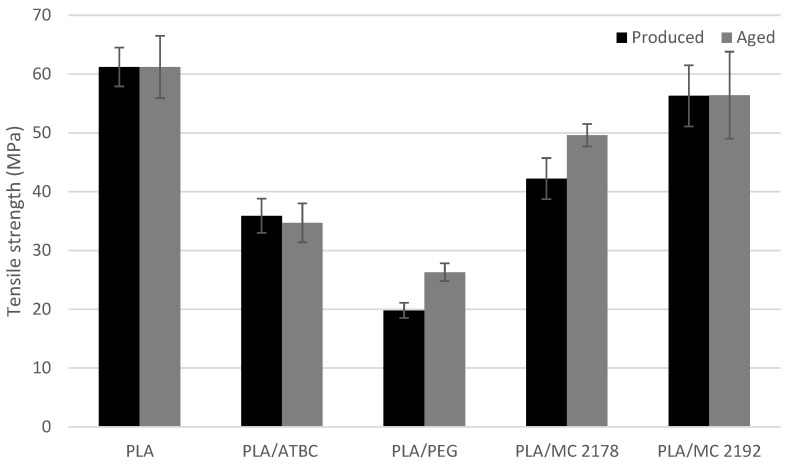
The evaluated results of tensile strength for as-produced and aged PLA films.

**Figure 3:**
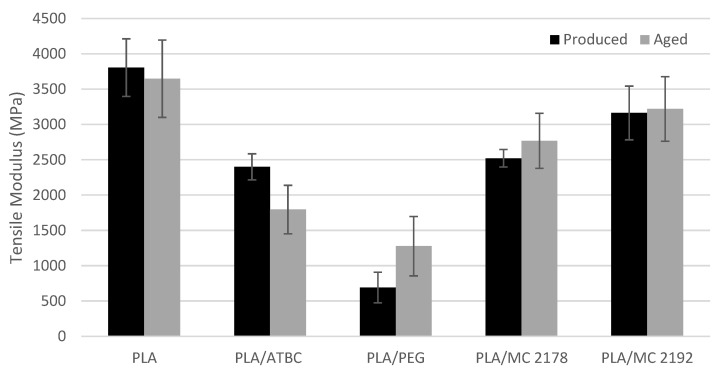
The evaluated results of tensile modulus for as-produced and aged PLA films.

The results of the mechanical properties of as-produced and aged plasticized PLA films are shown in Figure 1, Figure 2 and Figure 3. The plasticizers low molecular weight allows them to occupy intermolecular spaces between polymer chains. Due to this, they cause a reduction in energy for molecular motion, and the chain mobility is increased [10]. The influence of plasticizer on mechanical and rheological properties of PLA films will reflect its molecular weight and chemical composition (interaction between plasticizer and macromolecular chains of PLA). Consequently, different impacts on mechanical properties, such as fracture resistance and ductility enhancement, could be expected from the incorporation of plasticizers with different molecular weights and chemical compositions. The addition of 15 wt. % ATBC and PEG plasticizers to PLA evoked a significant increase in elongation at break under uniaxial loading of films. The 82% (PLA/ATBC) and 57% (PLA/PEG) increase in elongation were observed when compared to neat PLA. Courgneau et al. [14] reported equal efficiency of PLA/ATBC and PLA/PEG films at lower concentrations than 13 wt. %. However, at higher plasticizer contents, a higher elongation was found for PLA/ATBC. Rapa et al. [31] and Gálvez et al. [32] also found higher plasticizing efficiency of ATBC compared to PEG plasticizers at a higher content. The elongation at break of 250% (PLA/ATBC) and 140% (PLA/PEG) was achieved by the incorporation of 20 wt. % of plasticizer. The molecular weight influence of PEG on PLA plasticizer (15 wt. % concentration) is presented in the study by Darie-Niţă et al. [12]. The higher molecular weight of PEG 2000 and PEG 4000 evoked lower ductility changes in PLA (elongation lower than 5%). The influence of molecular weight on mechanical properties could be confirmed by the results of dicarboxylic acid-based plasticizers MC 2178 and MC2192. Only slight changes in elongation were observed for MC 2192, which had the highest molecular weight among those used. On the contrary, a significant enhancement in elongation (47%) was found for the PLA plasticized with MC 2178, which has the same chemical composition and a lower molecular weight. The low plasticizing efficiency of MC 2192 further evoked only a low decrease in tensile strength and modulus. Significant decreases in both properties were observed for MC 2178. The 19% decrease in tensile strength and 34% decrease in tensile modulus were evaluated for PLA/MC 2178. On the other hand, the incorporation of ATBC and PEG plasticizer into the PLA evoked significant changes. The 41% decrease in tensile strength and 37% decrease in tensile modulus were evaluated for ATBC-plasticized films. PEG-plasticised PLA films showed an even higher decrease in tensile strength (68%) and modulus (82%). Similar results were reported by Courgneau et al. [14], Rapa et al. [31], Gálvez et al. [32], and Greco et al. [33]. 

The results of the mechanical properties of aged films were compared to as-produced ones, and no changes in neat PLA films could be found. However, the differences in results of as-produced and aged plasticized PLA films could be observed. The aged PLA films plasticized with ATBC showed a significant decrease in elongation (57%), a considerable decrease in tensile modulus (25%), and minimal changes in tensile strength (4%). This drop could be ascribed to structural changes, the degradation process (chain scission), and the migration of plasticizer. The chain scission evoked a reduction in intermolecular forces and increased the mobility of the system. Consequently, tensile strength and modulus decreased. Furthermore, the enhancement of brittleness, and decrease in elongation and viscosity, is characteristic of the degradation process. The migration of ATBC, due to the lower content of plasticizer, could cause the ductility and fracture resistance to decrease. On the contrary, tensile strength, modulus, and viscosity will increase [34]. Regarding the stated results, the degradation (chain scission) could be ascribed as the primary reason for the change in properties of aged PLA/ATBC films. This confirmed the result of Rapa et al. [31,35], where a low migration of ATBC at 20 wt. % in PLA was observed. The greatest changes in mechanical properties were found for aged PLA/PEG films. The elongation at break drop on the level of neat PLA. The tensile modulus and strength increased by around 85% and 33%, respectively. The migration of plasticizer could be ascribed as the major reason for these changes. A similar dependence was reported in several studies. Hu et al. [36,37] observed a significant increase in tensile modulus and strength, and a decrease in fracture strain of plasticized PLA with 30% of PEG after 720 h ageing under ambient conditions (23 °C, 50% RH). Also, the results of Kodal et al. [16] are in agreement with our conclusions. The changes in mechanical properties of aged, plasticized PLA (one year at the ambient condition) were considerable at a content of PEG higher than 5 wt. %. The influence of molecular weight on changes in mechanical properties is also presented in this study. Greater changes were achieved using plasticizers with lower molecular weight (PEG 400) than plasticizers with higher molecular weight (PEG 8000, PEG 35000). The aged PLA films with the dicarboxylic acid-based plasticizers did not show, in the higher molecular weight variant (MC2192), changes in tensile modulus, strength, or elongation at break. A slight increase in tensile modulus and strength, as well as a decrease in elongation, was noticed for dicarboxylic acid-based plasticizer with lower molecular weight (MC2178). However, the differences are, with respect to levels of standard deviations, very low. Consequently, the presumption of migration or macromolecular scission could not be declared.

### 3.2. Rheological Properties

The evaluated rheological properties (MVR) of as-produced and aged PLA films are introduced in Table 3. All the plasticizers showed, after production, enhancement in flowability of PLA. An enormous increase (221-fold enhancement of MVR) was observed in PLA films plasticized with PEG. PEG is a plasticizer with very low viscosity. On the contrary, the plasticized PLA films with dicarboxylic acid-based plasticizer MC 2192, which has the highest molecular weight among those used, showed relatively small changes (1.48 times enhancement of MVR). The MVR of PLA plasticized with the lower molecular weight variation (MC2178) was around 15% higher. The incorporation of ATBC plasticizer ensured 2.2 times enhancement in MVR of PLA.

No changes in MVR of as-produced neat PLA films and aged ones were noticed. As in mechanical properties, the significant changes in rheological properties (MVR) were evaluated for plasticized PLA films. Also, for rheological properties, the highest changes showed PLA films containing PEG plasticizer. The MVR decreased by 47% after ageing. This result confirmed our conclusion (Section 3.1), where a migration effect of PEG plasticizer was ascribed as a major reason for changes in mechanical properties. The lower content of plasticizer causes lower enhancement of chain mobility of PLA, which leads to a decrease in rheological properties (flowability). The aged PLA/ATBC films showed the opposite dependence. A 15% increase in MVR was found. The chain scission could be due to a decrease in intermolecular forces ascribed to this enhancement. Also, this result is in accordance with the previous conclusion where the chain scission was presumed as a major reason for property changes in aged PLA/ATBC films. Furthermore, a significant increase in MVR was found for aged PLA films plasticized with dicarboxylic acids (MC2178 and MC2192). Therefore, the chain scission is a very important effect that has significant influence on properties of these aged films.

### 3.3. Differential Scanning Calorimetry (DSC)

**Figure 4:**
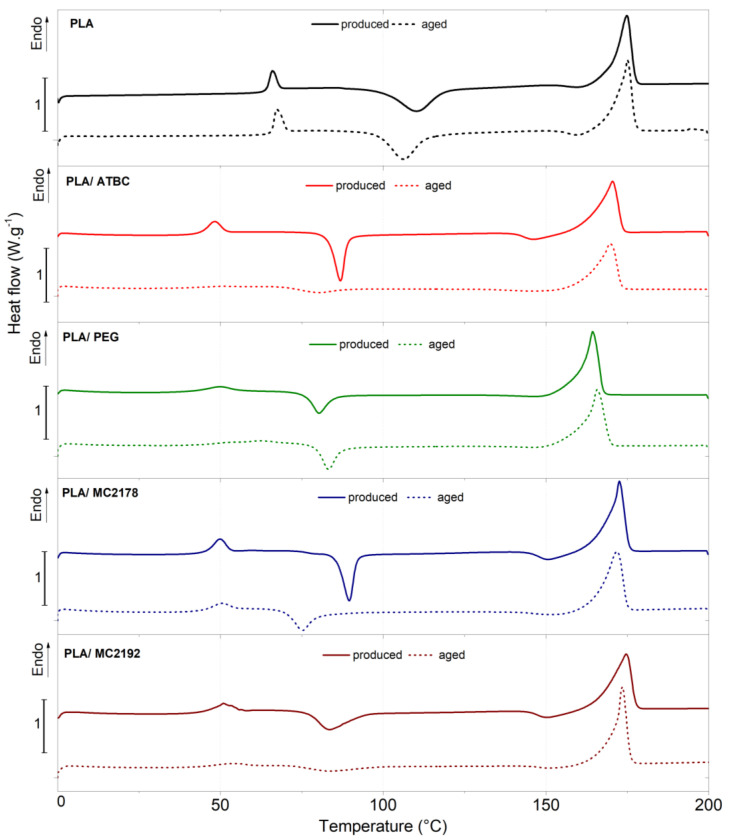
DSC curves of neat and plasticized PLA films after production and accelerated ageing.

The results of the first non-isothermal heating of as-produced and aged PLA films are shown in Figure 4 and in Table 4. The glass transient temperatures were used to neglect the technological processing aspects evaluated from the second heating cycle. A shift in transient temperatures is evident from the estimated results of as-produced plasticized PLA films. The glass transition temperatures of PLA films plasticized with ATBC decreased compared to neat PLA film by about 18 °C, cold crystallization temperature by about 23 °C, and melt temperatures by about 3 °C. A shift of about 23 °C at the glass transition temperature, 25 °C of cold crystallization temperature, and about 6 °C of melt temperature was noticed for PLA/PEG films. Also, Rapa et al. [31], Galvez et al. [32], and Farah et al. [38] reported similar temperature changes for PLA/ATBC and PLA/PEG. In several studies [12,39], the lower shift of transient temperatures was evaluated for increasing molecular weight of the PEG plasticizer. The influence of molecular weight is evident if the transient temperatures of dicarboxylic acid-based plasticizers MC2178 and MC2192 are compared. The plasticizer MC2192, having the highest molecular weight among those used, showed lower changes in glass transition (about 7 °C) and no changes in melt temperatures. Nevertheless, a significant decrease in cold crystallization (about 21 °C) was evident from the evaluated results. The plasticizer MC2178, with the same chemical composition and a lower molecular weight, showed a significantly higher decrease in these values. The glass transition temperature decreased by about 20 °C, cold crystallization by 20 °C, and melt temperature by 2 °C. The appearance of a double peak at melting temperature was reported by Rapa et al. [31] and Greco et al. [33] for PLA films plasticized with PEG and ATBC. Also, in our previous study [40], we reported the presence of a bimodal peak for PLA films with 10 wt. % of ATBC. Wu et al. [41] ascribed the dual melting peak to the formation of different crystalline structures (α and α’crystals). However, only the single melting peaks were noticed for all produced films. This could be caused by a higher content of plasticizers (15 wt. %), which will evoke higher chain mobility enhancement during processing, by the used type of PLA. Luminy L130, which was used in this experiment, has a higher content of L-isomer (99% L-isomer stereochemical purity) and higher crystallization ability than Ingeo 3001D (95 wt. % of L- lactide), which was used in the previous experiment.

From the evaluated results, it is evident that all plasticizers evoked, due to an increase in chain mobility, further structural changes. The highest crystallinity degree enhancements (crystallinity degree about 30%) were found for PLA/PEG films. On the contrary, the lowest enhancement of crystallinity (crystallinity degree of about 13%) was achieved for the highest molecular weight dicarboxylic acid-based plasticizer MC 2192. Also, PLA/ATBC films showed only a minor enhancement in crystallinity degree (about 14%). The dicarboxylic acid-based plasticizer MC2178 evoked, due to lower molecular weight (when compared to MC2192), greater improvement of PLA crystallinity (about 18% crystallinity degree).

No significant changes in transient temperatures between as-produced and aged neat PLA films have been found. Further, no change in crystallinity degree has been observed. Also, aged PLA films plasticized with ATBC did not show any considerable shift in transient temperature that made it possible to make a valuable statement. Nevertheless, significant changes in structure could be assumed for PLA/ATBC films after ageing. These structural changes were not evaluated via the degree of crystallinity but by the enthalpy changes due to the migration of plasticizer that could change weight content in PLA films. The cold crystallization and primary melt crystallization enthalpy markedly decreased in PLA/ATBC films after ageing. Consequently, the increase in enthalpy balance ∆H (Equation (3)) and enhancement of crystallinity degree could be assumed. In contrast, aged PLA films plasticized with PEG did not show any changes in enthalpies. Therefore, any valuable structure changes are not predicted. This could be ascribed to the high crystallization kinetics of PLA/PEG films, which already evoked a high level of structure order (degree of crystallinity) after production. The lower crystallization kinetics of PLA/ATBC films were stimulated by the supplied energy in the form of radiation during ageing and caused further structure order changes. Also, for as-produced PLA films that contain dicarboxylic acid-based plasticizer MC 2192, the lower crystallization kinetic (lower degree of crystallinity) was evaluated. However, due to higher molecular weight, only small changes in enthalpies were evaluated after accelerated ageing. Consequently, low structural changes could be assumed. Even lower changes in enthalpies were evaluated for MC 2178 plasticizer (lower molecular weight than MC 2192). This could be ascribed, as for PLA/PEG films, to the higher crystallization kinetics of PLA/MC2178 films during processing. If the glass transition temperatures of aged PLA films plasticized with PEG and MC2178 are compared to as-produced ones, the significant shifts are obvious. The glass transient temperatures increased by about 9 °C for PLA/PEG films and about 3 °C for PLA/MC 2178 films. The increased glass transient temperature is probably caused by the migration of plasticizers. The lower content of plasticizers in PLA will cause increased intramolecular bonding forces. On the contrary, any valuable shifting of glass transition was not evaluated for PLA/MC2192 films, as well as for PLA/ATBC films. Consequently, a low migration assumption could be declared.

The as-produced and aged neat PLA film exposed to thermophilic composting showed similar results (Table 4, Figures S1 and S2). After the first 14 days of composting, changes in transient temperatures had not been observed. However, the conditions in the thermophilic compost environment evoked the elimination of cold crystallization enthalpy, enhancement of melting enthalpy, and, consequently, enhancement of crystallization degree. Also, from the visual appearance of the films, it was evident that the thermophilic composting environment caused significant structural changes (loss of transparency). Further, 14 days of composting caused about a 6 °C decrease in the glass transient temperature, about 10 °C decreases in melt temperature, the elimination of primary crystallization enthalpy, and another enhancement in crystallinity degree. With regard to the increase in crystallinity degree, it can be stated that the further enhancement of crystallinity could be related to the degradation of the amorphous phase due to the hydrolysis. The degradation of amorphous parts (shortening of macromolecular chains within the amorphous region) could be ascribed to the reason for the decrease in transient temperatures. Jimenez et al. [42] reported that hydrolytic chain cleavage proceeds preferentially in the amorphous regions and leads, consequently, to an increase in crystallinity degree. The as-produced and aged PLA films plasticized with ATBC showed a slight increase in glass transient temperature (about 4 °C) after the first 14 days of composting. This phenomenon could be related to the migration and release of ATBC plasticizer in the environment. Similar to neat PLA films, the elimination of cold crystallization enthalpy and obvious increase in enthalpy balance (∆H) was evaluated for as-produced and aged PLA/ATBC films. This increase could be ascribed to the aforementioned structure order changes, degradation of amorphous phase, or the migration of plasticizers. Further, 14 days of composting of as-produced and aged PLA/ATBC films evoked another enhancement of enthalpy balance (∆H). However, due to decreasing content in the amorphous phase, it was not possible to evaluate glass transient temperature. The as-produced PLA/PEG films showed even higher shifting of transient temperatures after the first 14 days of composting. The glass transient temperature was increased by about 11 °C and melt temperature by about 5 °C. The elimination of cold crystallization, primary melting enthalpies, and enhancement of enthalpy balance (∆H) was further evaluated. The next 14 days of composting did not evoke any significant changes. This could mean that the main process of migration and changes in structural order were already finished. No enthalpy changes could also mean low degradation of the amorphous part. Therefore, it was not possible to evaluate the glass transient temperature for the aged PLA/PEG. However, minimal changes in melting temperature within 28 days of composting were evaluated. Therefore, compared to as-produced PLA/PEG films, a lower release of PEG into the compost environment could be assumed. The reason for this could be the high migration tendency of PEG plasticizer evoked by the process of ageing. The aged PLA/PEG films consequently contained a lower content of plasticizer that could be released during composting. On the contrary, for as-produced PLA/PEG films, a slight increase in enthalpy balance (∆H) was observed. The supposed low-level of releasing of plasticizer and high structure order level (minimal structural changes) could mean more intensive degradation. Also, for the dicarboxylic acid-based plasticizers, it was not possible to evaluate the glass transition temperatures for composted films. Nevertheless, from the shifting of melting temperature and evaluated enthalpies (∆H), some differences are obvious. Similar to PLA/ATBC and PLA/PEG films, the increase in melt temperature of as-produced PLA/MC 2178 films were evaluated after the first 14 days of composting. Consequently, some release of plasticizer into the compost environment could be assumed. Further, the elimination of cold crystallization and enhancement of enthalpy balance (∆H) was evaluated. The following 14 days of composting evoked another decrease in melt temperature and enhancement of enthalpy (∆H). Regarding the previous summary, a more intensive degradation (chain scission) of amorphous parts than for PLA/PEG films could be assumed. The composted, aged PLA/MC 2178 films did not show any significant changes in melt temperature within the first 14 days. This result could also be ascribed (as well as for PLA/PEG films) to some migration tendency of MC2178 of plasticizer, evoked by the ageing process. However, after the next 14 days of composting, the PLA/ MC 2178 films showed a decrease in melt temperature and further enhancement of enthalpy (∆H). Therefore, for aged PLA/MC2178, also, the degradation of the amorphous phase could be assumed to be the main aspect of this event. The as-produced and aged composted PLA films that contained plasticizer MC 2192 showed, after the first 14 days of composting, the highest decrease in melting temperature and enthalpy (∆H) enhancement. Regarding this, and low migration/releasing tendency of MC2192 plasticizer, the highest degradation rate could be assumed for these films.

### 3.4. Thermogravimetric Analysis (TGA)

**Figure 5:**
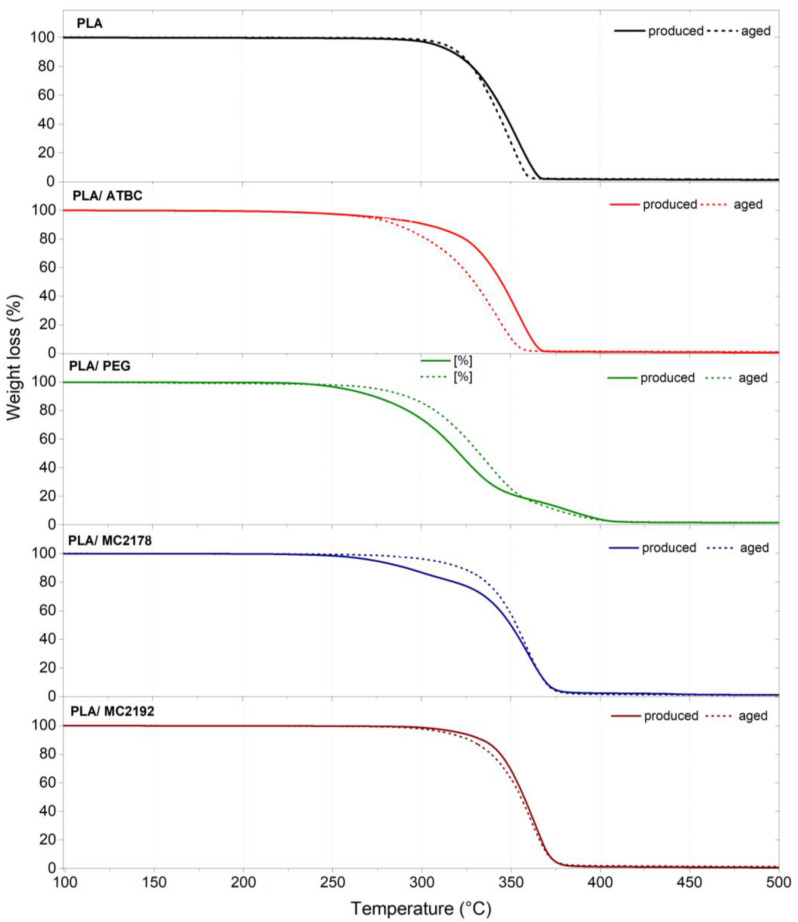
TGA curves of neat and plasticized PLA films after production and accelerated ageing.

The results of TGA are shown in Figure 5 and summarized in Table 5. Several studies reported significant decreases in thermal stability using ATBC and PEG plasticizers in PLA [10,11,32,43,44]. However, only a low decrease in thermal stability (initial decomposition temperature T5%) was observed for as-produced PLA/ATBC films. The reason could be different types of PLA (molecular weight, L and D isomer content, etc.) and their interaction with ATBC. Furthermore, applied technology (compression molding, extrusion, casting, etc.) as well as processing conditions, could also be contributing factors. Also, the addition of dicarboxylic acid-based plasticizer MC2192 to PLA films evoked only a low decrease in thermal stability. The incorporation of MC2178 plasticizer with the lower molecular weight prompted a higher decrease in thermal stability. The initial decomposition temperature (T5%) decreased by about 16 °C when compared to neat PLA. The highest change in thermal stability was observed for PLA films plasticized with PEG. The initial decomposition temperature (T5%) decreased by about 53 °C. The evaluated result of thermal stability did not show any considerable changes between as-produced and aged neat PLA films. Furthermore, minimal differences were evaluated between as-produced and aged PLA films plasticized with MC2192. The aged PLA/ATBC films showed approximately a 9 °C decrease in initial decomposition temperature (T5%). This result could be ascribed to an event of chain scission that evokes a decrease in intramolecular forces. On the contrary, an increase in initial decomposition temperature (T5%) was found for aged PLA/PEG and PLA/MC2178 films. The initial decomposition temperature (T5%) increased by about 10 °C for PLA plasticized with MC 2178 and about 37 °C for PLA plasticized with PEG. The increase in thermal stability is, as well as a shift in transient temperatures and decrease in viscosity (MVR), evoked by the migration of plasticizer. Consequently, the previously-stated presumption (Section 3.2 and Section 3.3) about the tendency of migration for these two plasticizers could be confirmed.

The thermophilic composting process of as-produced and aged neat PLA films showed major changes in thermal stability, similar to changes in transient temperatures and enthalpies (Section 3.3), after 28 days of composting (Table 5, Figures S3 and S4). About a 20 °C decrease in initial decomposition temperature (T5%) was found for as-produced neat PLA films, and about 28 °C decrease for aged ones. Consequently, considerable degradation (hydrolytic chain scission) could be assumed. On the other hand, as-produced and aged PLA/ATBC films showed considerable changes in thermal stability even after 14 days of composting. The initial decomposition temperature (T5%) of as-produced PLA/ATBC films decreased by about 17 °C and, for aged PLA/ATBC films, about 10 °C. Also, the further 14 days of composting evoked another considerable decrease (about 18 °C) in initial decomposition temperature (T5%). A relatively low decrease in initial decomposition temperature (about 10 °C) was observed for as-produced PLA/PEG films after the first 14 days of composting. Another 14 days of composition did not evoke any significant changes in thermal stability. Therefore, a lower level of degradation (chain scission) than for neat PLA and PLA/ATBC films could be assumed. Nevertheless, it is important to mention that the results of thermal stability are also be influenced by the migration of plasticizer. The aged PLA/PEG films showed a higher decrease in thermal stability. The initial decomposition temperature (T5%) decreased by about 16 °C after 14 days of composting and by about 46 °C after 28 days of composting. The low changes in thermal stability within 28 days of composting were evaluated for as-produced PLA films plasticized with dicarboxylic acid-based plasticizer MC2178. The ageing process of PLA/MC2178 films evoked obviously higher changes. The initial decomposition temperature (T5%) decreased by about 16 °C at the first 14 days of composting and about 36 °C after 28 days. Consequently, a higher degradation (chain scission) of aged PLA films plasticized with MC2178 and PEG than for as-produced ones could be assumed. The highest decrease in initial decomposition temperature (highest degradation) was observed for composted PLA films plasticized with MC2192. The 14 days of thermophilic composting evoked about a 10 °C decrease in initial decomposition temperatures (T5%) and, after 28 days of composting, decreased by about 56 °C. No significant differences were observed between as-produced and aged films.

### 3.5. Scanning Electron Microscopy (SEM) Analysis

**Figure 6:**
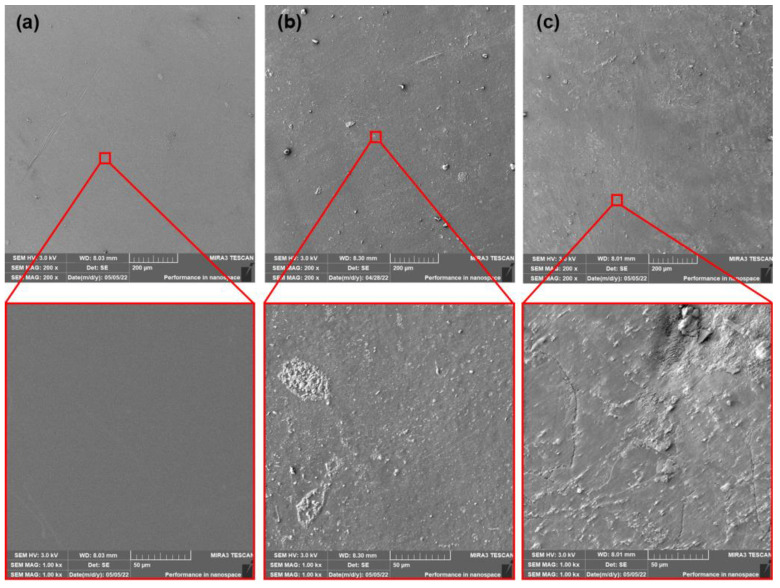
SEM images of as-produced neat PLA films (**a**) at initial state and after (**b**) 14 days of thermophilic composting, and (**c**) 28 days of thermophilic composting.

**Figure 7:**
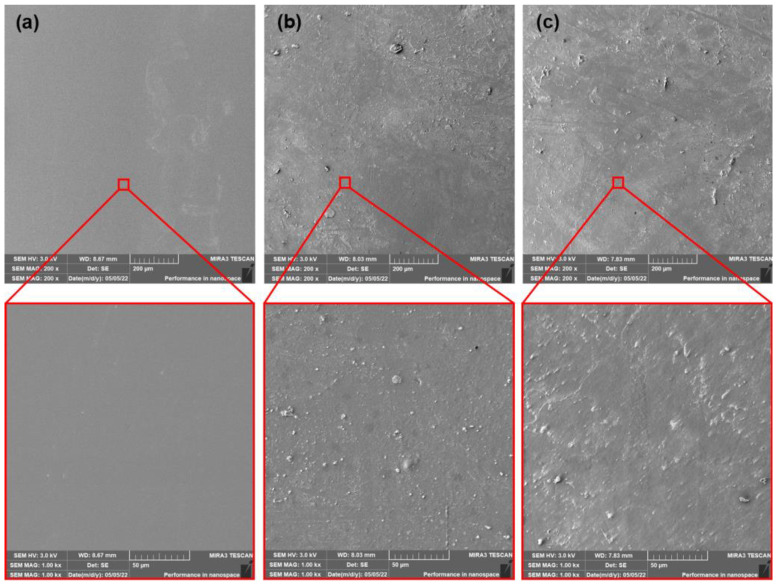
SEM images of aged neat PLA films (**a**) at initial state and after (**b**) 14 days of thermophilic composting, and (**c**) 28 days of thermophilic composting.

**Figure 8:**
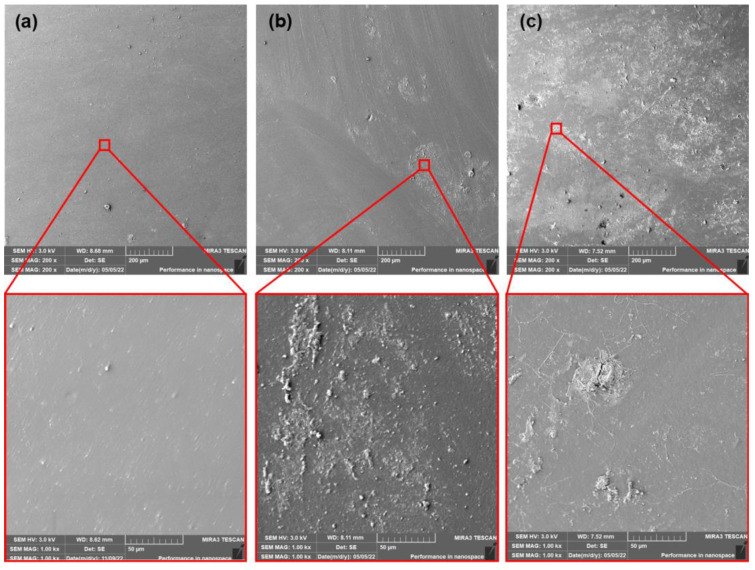
SEM images of as-produced PLA/ATBC films (**a**) at initial state and after (**b**) 14 days of thermophilic composting, and (**c**) 28 days of thermophilic composting.

**Figure 9:**
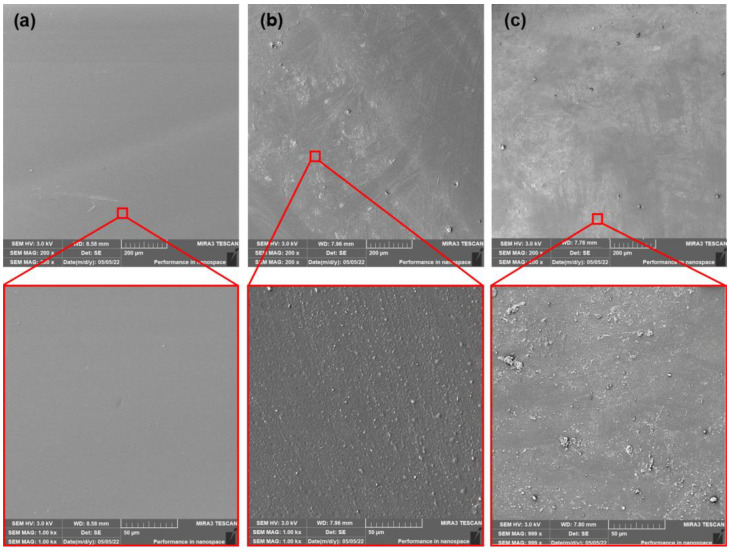
SEM images of aged PLA/ATBC films (**a**) at initial state and after (**b**) 14 days of thermophilic composting, and (**c**) 28 days of thermophilic composting.

The SEM images of surfaces of neat, as-produced PLA films and films after thermophilic composting (14 and 28 days) are shown in Figure 6. The PLA samples after ageing and subsequent thermophilic composting are shown in Figure 7. The neat PLA films showed smooth surfaces after the production and accelerated ageing. The exposition of as-produced films to thermophilic composting evoked only a slight increase in the roughness of their surface after the first 14 days. A significant change in thermal properties after 28 days of composting was observed. However, a very low enhancement of surface erosion could be seen from SEM images. This could mean that mainly bulk degradation occurred. Arrieta et al. [45] reported similar results. Minimal surface erosion was observed within 28 days of lab thermophilic composting of PLA films. No significant erosion of the surface was observed for aged PLA films after 14 or 28 days of composting. The SEM images of surfaces of as-produced and aged PLA/ATBC films and PLA/ATBC films after thermophilic composting (14 and 28 days) are shown in Figure 8 and Figure 9.

**Figure 10:**
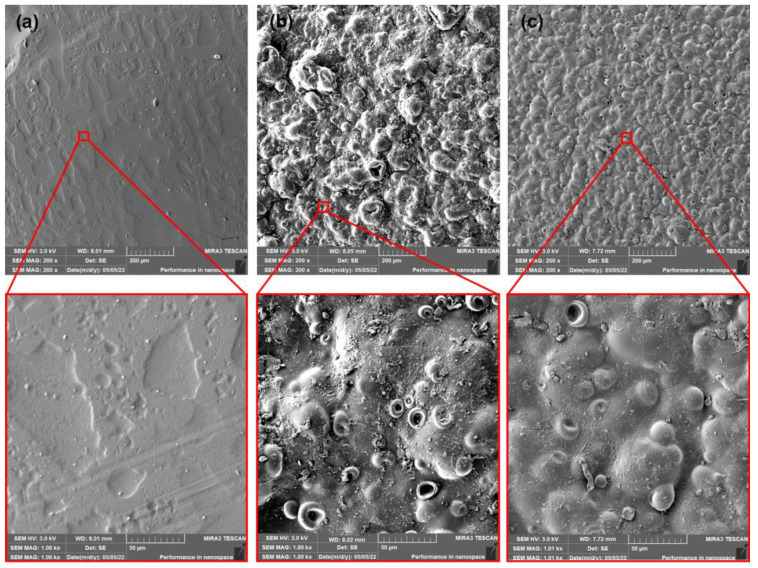
SEM images of as produced PLA/PEG films (**a**) at initial state and after (**b**) 14 days of thermophilic composting, and (**c**) 28 days of thermophilic composting.

**Figure 11:**
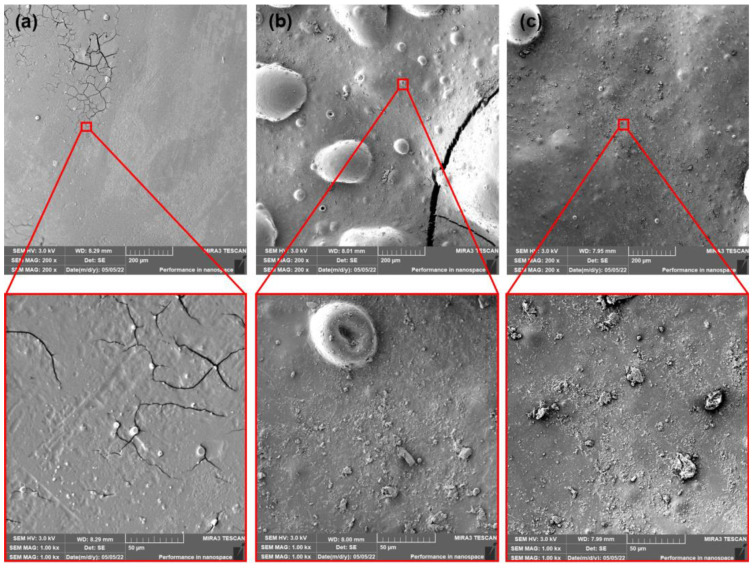
SEM images of aged PLA/PEG films (**a**) at initial state and after (**b**) 14 days of thermophilic composting, and (**c**) 28 days of thermophilic composting.

The incorporation of PEG plasticizer into PLA films evoked more evident changes. The surfaces of as-produced PLA and PLA/ATBC films were considerably smoother than as-produced PLA/PEG films (Figure 10). The appearance of the “lunar surface” morphology was characteristic of these films. Low viscosity and poor miscibility of PEG with PLA at higher concentrations [14] could be a reason for this event. The PEG plasticizer, during extrusion through the head, is released on the surface and limits the process of calandering (entrapping of plasticizer between rolls and films). The first 14 days of composting evoked enormous changes in surface roughness. The surface was very rugged with a large number of “craters.” The intensive release of water-soluble PEG plasticizer and the degradation of amorphous parts could be ascribed as the main phenomena of these changes. Another 14 days of composting did not evoke such dynamic changes. The level of roughness was similar to the first evaluated period. Regarding previous results (change in mechanical, rheological, and thermal properties), the ageing process evoked a significant migration of PEG plasticizer from PLA films. Therefore, an increase in brittleness could be expected. This assumption could be confirmed by the SEM surface images of aged PLA/PEG films (Figure 11). The appearance of cracks could be seen on the surface of aged films. Furthermore, due to the lower content of PEG plasticizer in PLA films, a lower level of release into the compost environment could be expected. Because of the lower level of roughness, a lower numbers of “craters” compared to as-produced films could be seen. This presumption could be confirmed by analysis of the surface images of aged PLA/PEG films after 14 days of composting. Despite being rough, a large amount of craterless surface could be seen after 28 days of composting. Thus, again, a minimal migration of PEG could be further expected.

**Figure 12:**
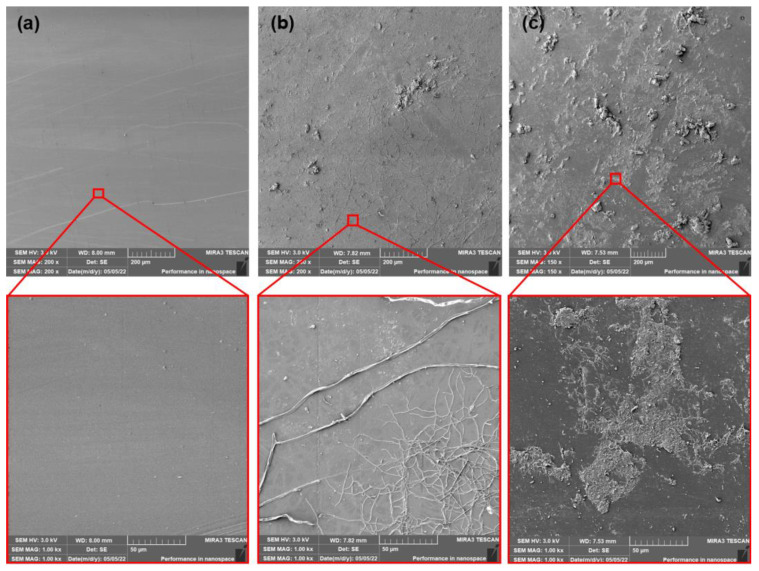
SEM images of as-produced PLA/MC 2178 films (**a**) at initial state and after (**b**) 14 days of thermophilic composting, and (**c**) 28 days of thermophilic composting.

**Figure 13:**
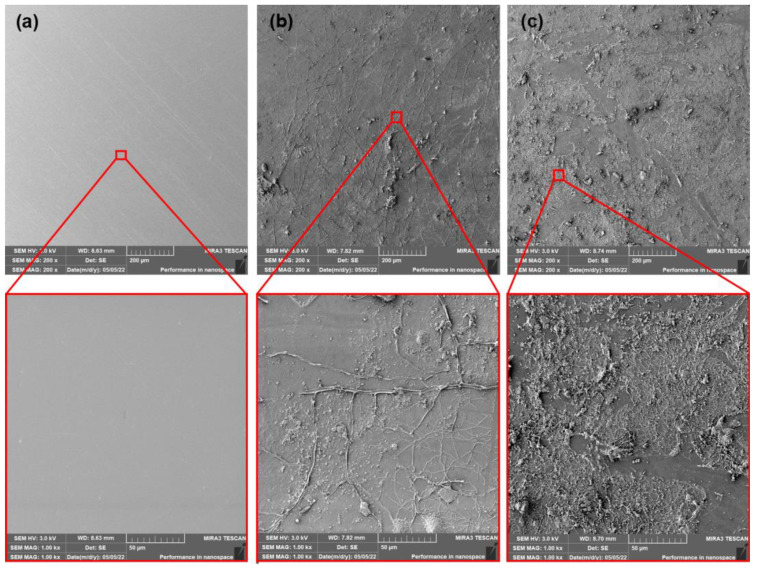
SEM images of aged PLA/MC2178 films (**a**) at initial state and after (**b**) 14 days of thermophilic composting, and (**c**) 28 days of thermophilic composting.

**Figure 14:**
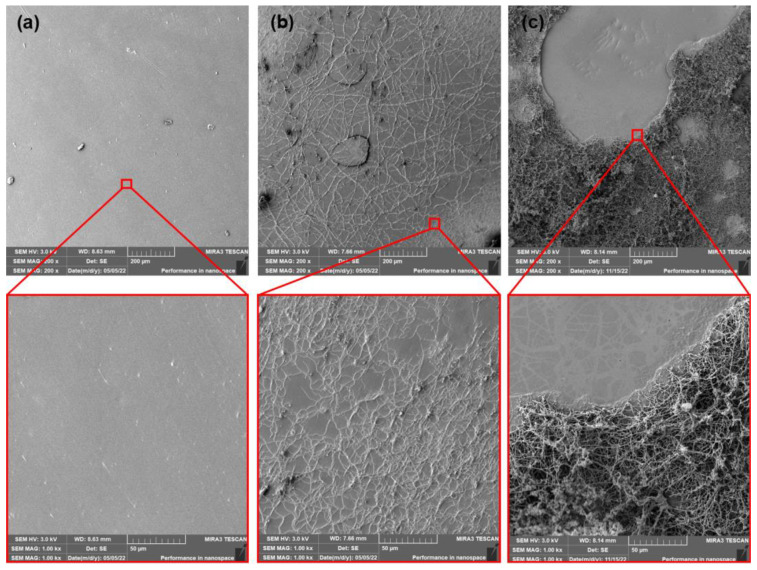
SEM images of as-produced PLA/MC2192 films (**a**) at initial state and after (**b**) 14 days of thermophilic composting, and (**c**) 28 days of thermophilic composting.

**Figure 15:**
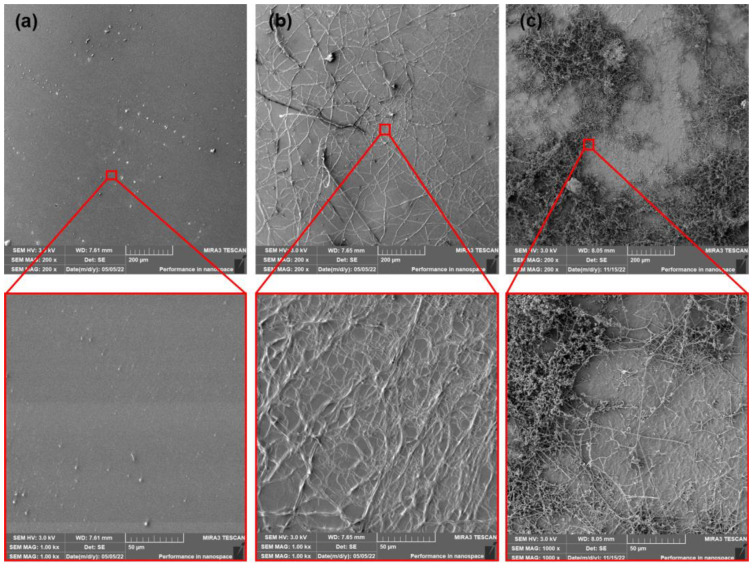
SEM images of aged PLA/MC2192 films (**a**) at initial state and after (**b**) 14 days of thermophilic composting, and (**c**) 28 days of thermophilic composting.

The PLA films that contained dicarboxylic acid-based plasticizer MC2178 showed (Figure 12 and Figure 13) smooth surfaces after production and ageing process. The first 14 days of composting did not evoke any significant changes in surface roughness. However, the initiation of algae fibres growth was observed. If the as-produced and aged PLA/MC2178 films are compared, a slightly higher algae fibres ratio is observed for the aged films. The differences after 28 days of composting are more obvious. The higher activity of algae fibres could evoke faster disturbance of the surface, which could increase the kinetics of disintegration, which further, would lead to biotic attack and biodegradation of films. An even higher ratio of algae fibres was observed for PLA films plasticized with MC2192 after the first 14 days of composting (Figure 14 and Figure 15). It is well known that the formation of lactic acid oligomers during chain scission of PLA increases the concentration of carboxylic acid end groups in the degradation medium. The catalytic action of these groups at their increasing content further results in a self-catalyzed and self-maintaining process [46]. Based on the increased content of carboxylic acid groups in both PLA/MC2178 and PLA/MC2192 when compared to other plasticizers, it could be assumed that they, during degradation, stimulate biotic attack. Furthermore, Ren et al. [22] reported that water molecules easily diffuse into amorphous regions, and these regions are also easily assimilated by microorganisms. Consequently, the easy assimilation of algae fibres could also be assumed. Another exposition (14 days) of PLA/MC2192 films to composting further enhanced the algae fibres activity. The hydrolytic degradation and microbial/enzymatic activity caused the flaking of fragments. Differences between as-produced and aged films were observed within the 28 days of composting.

### 3.6. Fourier Transform Infrared Spectroscopy (FT-IR)

**Figure 16:**
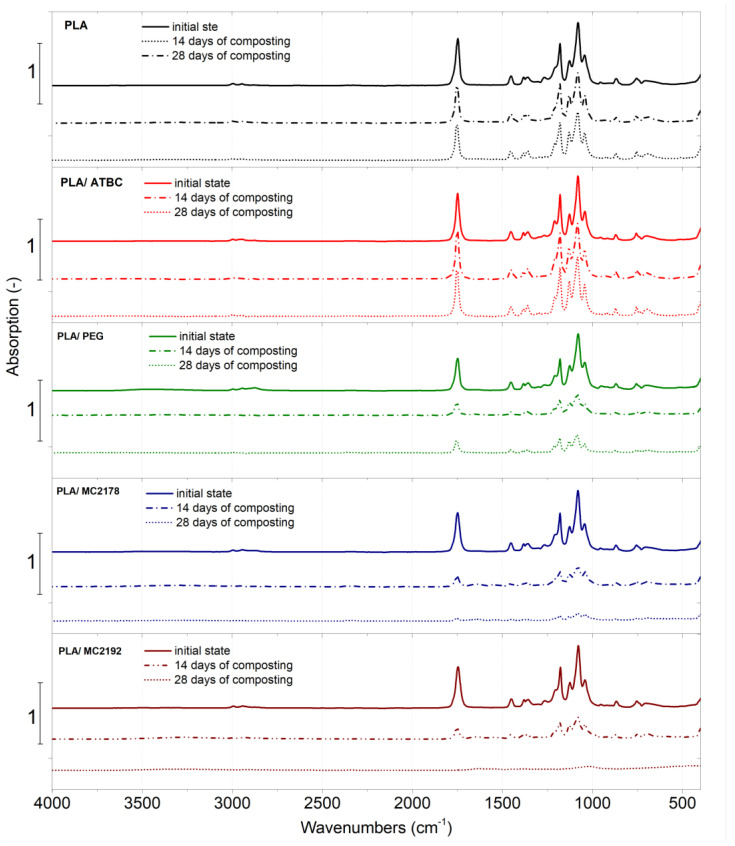
FTIR spectra of as-produced PLA films at initial state and after 14 days and 28 days of thermophilic composting.

According to the evaluated results, no significant differences in the ATR-FT-IR spectra between as-produced and aged PLA and PLA-plasticized films were observed. Consequently, assessed and discussed are only the as-produced and composted ones (Figure 16). The ATR-FT-IR spectra of aged films are added in supplementary materials (Figures S5 and S6). Typical absorption bands for PLA, corresponding to the C=O stretching of ester groups at 1747 cm^−1^, with asymmetric and symmetric CH_3_ stretching at 2995 cm^−1^ and 2945 cm^−1^, the C-O stretching bands of –CH–O– at 1180 cm^−1^, and –O–C=O groups at 1127 cm^−1^, 1080 cm^−1^, and 1043 cm^−1^, respectively, were observed [15,47,48,49]. Moreover, similar to the work of Zaidi et al. [50], bending frequencies for CH_3_ were identified at 1452 cm^−1^, 1382 cm^−1^, and 1359 cm^−1^. As well, bands related to the C=O double-bound around 700 cm^−1^ were further observed. When comparing the spectra of as-produced neat PLA films and PLA films plasticised with ATBC at the initial state and within 14 and 28 days of composting, no significant differences were noted. On the contrary, the spectra of as-produced PLA films plasticised with PEG and dicarboxylic acid-based plasticizers (MC2178 and MC2192) showed an obvious decrease in absorption intensity after 14 days of composting. According to results of Vasile et al. [51], the decrease in peak ratio declares chemical changes that could be evoked by the process of hydrolytic degradation (chain scission). Another 14 days of composting did not evoke any considerable changes in ATR-FT-IR spectra for PLA/PEG films. However, the dicarboxylic acid-based plasticizers in PLA showed another significant decrease in peak intensity. Especially, composted PLA films with MC2192 plasticizer showed a very low peak intensity after 28 days of composting. Consequently, extensive degradation could be, for these films, assumed.

**Figure 17:**
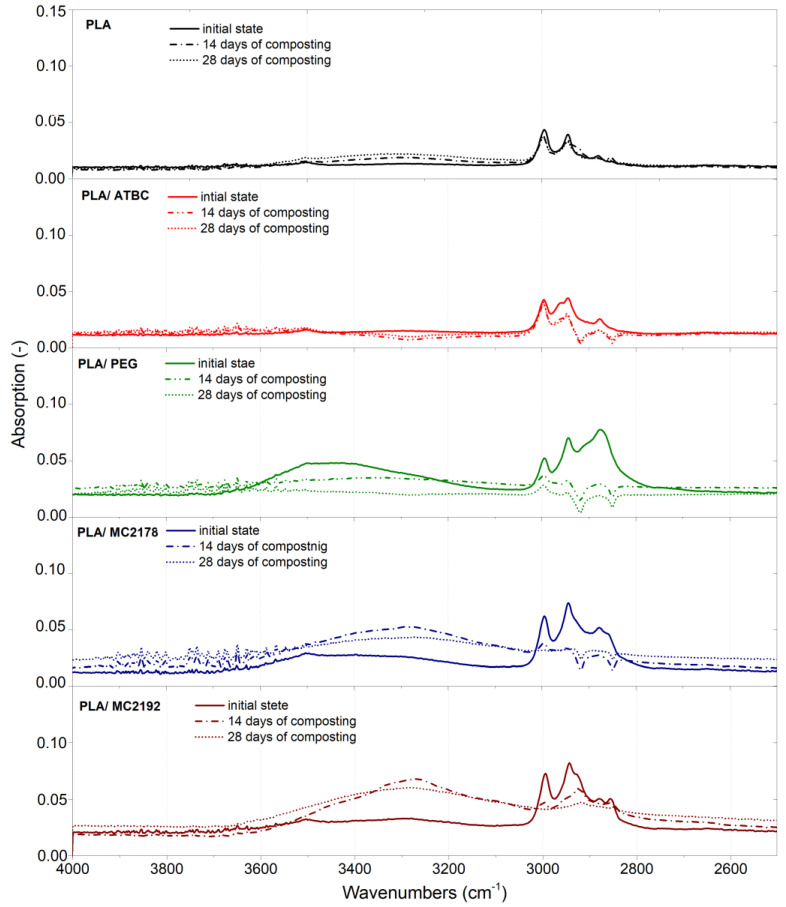
FTIR spectra of as-produced PLA films at initial state and after 14 days and 28 days of thermophilic composting that indicate hydroxyl bands.

Oliveira et al. [52] and Kammoun et al. [53] reported that, during PLA degradation, the appearance of hydroxyl bands around 3400 cm^−1^ indicates the occurrence of hydrolysis degradation. Consequently, the comparison of changes in peak intensity at this area has been separately evaluated in Figure 17. Only minimal changes in hydroxyl bands were observed for neat PLA films and PLA films plasticized with ATBC within 28 days of composting. PEG is a water-soluble plasticizer with polar (-OH) groups. Therefore, the increase in hydroxyl bands was evaluated by the incorporation of this plasticizer into PLA films. Also, Rafie et al. [39] and Darie-Nita et al. [12] observed the enhancement of hydroxyl bands around 3400 cm^−1^ if the PEG plasticizer was incorporated into PLA. According to the decreasing peak intensity of hydroxyl bands evaluated by the process of composting, the stated migration and release of plasticizer into the environment could be confirmed. Within the composting process, the significant increase in peak intensity of hydroxyl bands was for both dicarboxylic acid-based plasticizers, which were further observed. The rapid hydrolysis could consequently evoke faster disintegration, assimilation by microorganisms/enzymes, and faster biodegradation.

### 3.7. Degree of Disintegration of Composted PLA Films

**Figure 18:**
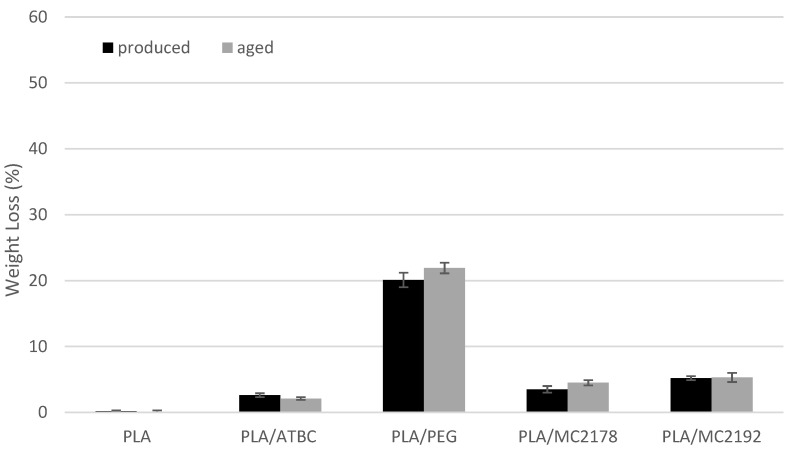
The weight loss of as-produced and aged PLA films after 14 days of composting.

**Figure 19:**
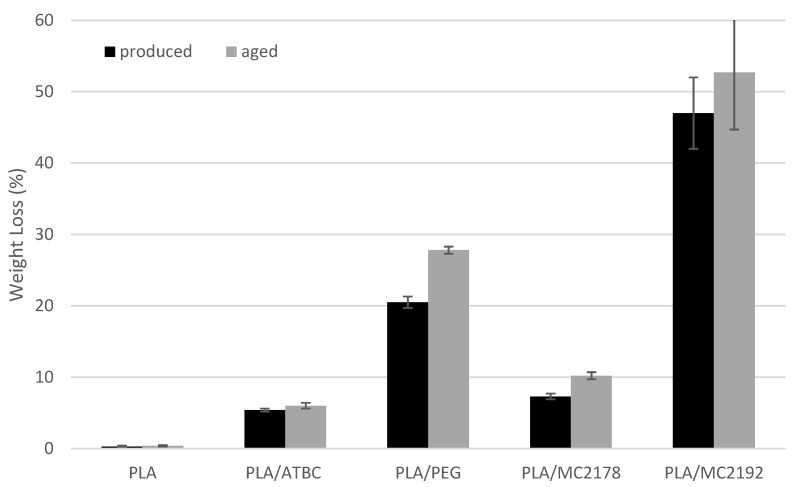
The weight loss of as-produced and aged PLA films after 28 days of composting.

The evaluated results of weight loss of as-produced and aged PLA films after 14 and 28 days of thermophilic composting are shown in Figure 18 and Figure 19. With respect to standard deviation, the as-produced and aged neat PLA films did not show any changes in weight after the first 14 days of thermophilic composting. Further exposition of as-produced and aged neat PLA films to the compost environment evoked considerable changes in thermal properties (DSC, TGA). However, any evident changes were obvious from SEM surface images. No considerable weight loss was found for these films after 28 days of composting. On the other hand, the as-produced PLA/PEG films showed about 20% weight loss after 14 days of composting. Regarding the previous conclusions, this weight loss could be ascribed mainly to the intensive release of plasticizer into the compost environment. Because the following exposition to the compost environment for another 14 days evoked only minimal changes in weight, the low tendency of as-produced PLA/PEG to disintegrate could be further assumed. Also, aged PLA/PEG films showed a high decrease in weight (about 21%) after the first 14 days of composting. Further composting of the aged PLA/PEG films evoked, in comparison to as-produced films, a higher weight decrease of about 28%. As it was introduced, during the aging process, some migration of PEG and minimal structure changes were found. Therefore, a lower ratio of releasing of plasticizer could be expected (SEM surface images, Figure 11). Consequently, the slightly higher decrease in the weight of aged PLA/PEG films was probably caused by higher disintegration rate. Significant differences in weight loss were evaluated for PLA films plasticised with dicarboxylic acid-based plasticizers within 28 days of composting. The higher molecular weight of MC2192 plasticizer evoked lower enhancement of crystalline structure of PLA films than for lower molecular ones (MC2178). PLA is polyester, where hydrolysis (bulk degradation) is one of the most critical factors for disintegration [54]. The water molecules easily diffuse through amorphous regions. Consequently, the PLA films plasticised with MC2192 should achieve a faster disintegration rate. The evaluated results for weight loss confirmed this presumption. The difference between the weight loss of as-produced films was, after 14 days of composting, low, being 3% weight loss for PLA/MC2178 films and 5% for PLA/MC2192 films. The only 4% enhancement of weight loss was after another 14 days of composting, evaluated for as-produced PLA/MC2178 films. However, about 47% weight loss was found for PLA films plasticised with MC2192. With respect to the evaluated standard deviation, we cannot state any relevant differences between as-produced and aged PLA/MC2192 films within 28 days of composting. Nevertheless, slightly higher increase in weight loss could be seen for aged PLA/MC2178 films than for as-produced ones. This could be caused (regarding the evaluated results of thermal analyses) by the releasing of plasticizer to the compost environment. Another reason could be higher microbial/enzymatic attack (the level of algae fibres observed on SEM surface images, Figure 12 and Figure 13) that evoke a faster process of disintegration. The as-produced PLA/ATBC films was characterized by low enhancement of degree of crystallinity (similar level as for PLA/MC2192 films) and low tendency to migration. Therefore, easier hydrolysis and faster disintegration were expected. However, the evaluated weight loss was only about 3% after 14 days of composting and about 5% after 28 days of composting. Consequently, the chemical composition of plasticizers must be stated as another crucial aspect that influences the disintegration rate. The importance of chemical composition of plasticizer could be confirmed from results of weight loss of aged PLA/ATBC films. During the ageing process of PLA/ATBC films, a significant increase in structure order was concluded. Nevertheless, the weight reduction in aged PLA/ATBC films was achieved within 28 days of composting at a similar level to the as-produced ones.

### 3.8. Biodegradability under Thermophilic Composting

**Figure 20:**
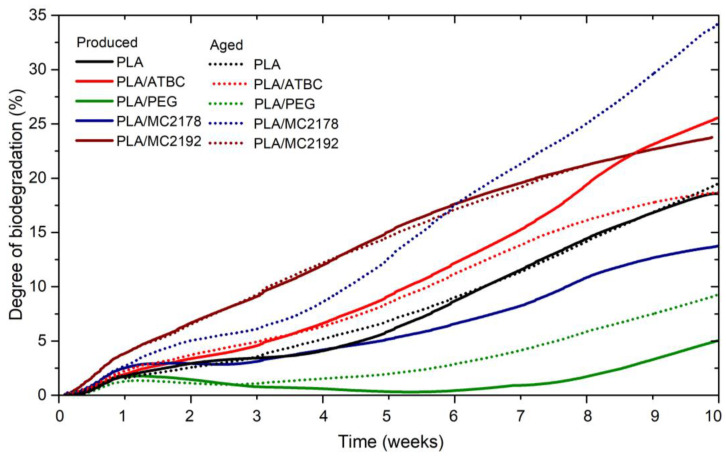
Biodegradation curves of as produced and aged PLA films under controlled thermophilic composting ISO 18455-1.

The evaluated biodegradation curves are shown in Figure 20. During the first 14 days of composting, similar courses and levels of biodegradation were evaluated for neat PLA films and PLA films plasticized with ATBC or dicarboxylic acid-based plasticizer MC 2178. Nevertheless, the PLA films plasticized with PEG and dicarboxylic acid-based plasticizer of higher molecular weight (MC2192) showed different biodegradation rates. The incorporation of PEG plasticizer caused a decrease in the biodegradation rate of PLA films. Polymer materials are microbially degradable in a two-step process. The first step consists of a reduction in the polymer chain into low molecular weight oligomers, dimers, and monomers that are short enough to be assimilated by microorganisms in the second step [54,55]. Consequently, the disintegration rate, which depends on the intensity of enzymatic and hydrolytic attack, has a crucial factor in biodegradation. As it was mentioned above, PEG is water-soluble hydrophilic plasticizer with polar groups (–OH). The hydrophilic character of plasticizer could increase the permeability of water and oxygen into the PLA films, as was presented in the study of Courgneau et al. [14]. Therefore, the incorporation of PEG plasticizer could increase the disintegration and biodegradation rate of PLA. However, Cao et al. [56] and Laboulfie et al. [57] reported that the water permeability depends on the molecular weight of the PEG plasticizer. The significantly lower water permeability was evaluated for films plasticized with a low molecular weight of PEG (PEG 300) compared to high molecular ones (PEG 4000). The reason for this could be hydrogen bonding between polar groups (-OH) of plasticizes and PLA. The high molecular weight PEGs might not be able to position themselves to create sufficient hydrogen bonds with polymers [58]. Other aspects that must be taken in consideration are the influence of plasticizer on microbial/enzymatic activity as well as the influence on the structure order (degree of crystallinity) of films. Kammoun et al. [53] evaluated the enhancement of the antibacterial activity of chitosan films if the PEG plasticizer was incorporated. However, it likely that the main reason for lower microbial activity (lower biodegradation rate) is the enhancement of crystallinity. As was introduced in Section 3.3, the low viscosity of PEG plasticizer evoked the highest enhancement of the degree of crystallinity from used plasticizers. The diffusion of water as well as enzymatic degradation take place primarily in the amorphous part. Consequently, slower biodegradation could be assumed.

The high molecular weight of dicarboxylic acid-based plasticizer MC2192 caused the lowest enhancement of crystallinity degree of PLA films among used plasticizers. Thus, the higher content of amorphous parts, the higher molecular weight of plasticizer (lower interaction bonds with PLA), and the self-catalyzed action caused by increasing content of carboxylic acid end groups could be the main reason for the higher disintegration (weight loss) and biodegradation level. Comparing the biodegradation of films after accelerated ageing, no significant difference was observed at the first 14 days of composting. The exception was only PLA/MC2178, where a slight increase in biodegradation level has been found for aged films. This result corresponds to SEM surface images (Figure 12 and Figure 13) where higher algae fibres activity was observed for aged films than for as-produced one. The biodegradation degree at 28 days of composing showed higher differences among the as-produced films. A degree of about 12% has been found for as-produced PLA/MC2192. The neat PLA films achieved about 4% biodegradation. The same results have been found for as-produced PLA/MC2178 films. Compared to neat PLA, a slight increase in the biodegradation degree was evaluated for as-produced PLA/ATBC films (biodegradation about 6%). ATBC is a water-insoluble plasticizer with better barrier properties against oxygen and water permeability than PEG [14,44]. Therefore, slower disintegration and biodegradation could be assumed. Nevertheless, the diffusion of water and oxygen in PLA film strongly depends on temperature. Courgenau et al. [14] evaluated extensive differences in water and oxygen diffusion of PLA/PEG and PLA/ATBC films at 25 °C. However, relatively low differences were evaluated at 38 °C. The increasing temperature has a similar effect as the plasticizing of PLA. The chain mobility is increased, and intermolecular forces decrease. Therefore easier diffusion of water and oxygen is achieved [14]. Similar to PLA/MC2192 films, the degree of crystallinity could be assumed to be the major factor for the higher biodegradation rate of PLA/ATBC. Compared to PLA/PEG films, a very small enhancement of the degree of crystallinity was observed for as-produced PLA/ATBC films. Consequently, easier diffusion of water molecules, easier assimilation of microorganisms, and faster disintegration and biodegradation were achieved. As-produced PLA/PEG films showed minimal disintegration and biodegradation degree. The differences in biodegradation between as-produced and aged films (expect PLA/MC2178) are still too small to make any valuable statement. However, at the end of the experiment (70 days), there are obvious differences between aged PLA/PEG and PLA/ATBC. Due to the intensive increase in degree of crystallinity for aged PLA/ATBC films, a considerably lower biodegradation rate than that for as-produced ones was achieved. The aged PLA/ATBC films achieved 19% biodegradation and as-produced ones reached up to 26% of biodegradation degree. According to the result of DSC analysis, no significant changes in crystallinity degree were observed for aged PLA/PEG films. Despite these findings, a slightly faster biodegradation rate is evident from the biodegradation curse, where about 5% enhancement of biodegradation level has been found for aged films. The reason for this could be a high tendency of PEG plasticizer to migration. Both Courgenau et al. [14] and Mariana et al. [44] reported that the migration of plasticizer evokes a decrease in hydrogen bonding between PLA/PEG and enhanced diffusion of water or oxygen. The highest level of biodegradation (34%) has been evaluated for aged PLA films plasticized with MC2178. On the contrary, only 14% biodegradation has been found for as-produced ones. This phenomenon could be ascribed to the high tendency to migration of MC2178 plasticizer, relatively small change in crystallinity degree during the aging process, and the chain scission of macromolecules which results in an easier water and oxygen diffusion process that evokes higher microbial/enzymatic attack. Another aspect could be the chemical composition and bonding between PLA and plasticizers. According to the SEM surface images, the dicarboxylic acid-based plasticizers that contain two carboxyl groups (–COOH) evoked evidently higher biotic activity. Consequently, faster disintegration and microbial degradation was achieved. The minimal differences in biodegradation were evaluated for as-produced and aged neat PLA films. Both films achieved about 18% biodegradation after 70 days of thermophilic composting. A slightly higher biodegradation rate (biodegradation of about 31% biodegradation within the same time period) was evaluated for as-produced neat PLA films in our previous study [40]. The faster biodegradation rate could be caused by the used PLLA. Luminy L130, used in this study, has a higher content of L-isomer (99% L-isomer stereochemical purity) than Ingeo 3001D (95 wt. % of L- lactide), which was used in the previous experiment. The stereochemical purity influences the structure order (Section 3.3) as well as biodegradation rate. Cadar et al. [59] reported some correlation between the composting and the level of biodegradation of PLA-based copolymers. The biodegradation of copolymers containing higher amounts of lactic acid was found to be faster than the biodegradation of copolymers containing smaller amounts. 

## 4. Conclusions

According to the evaluated results, the molecular weight and chemical composition of plasticizers are the main aspects that predetermine the future mechanical, rheological, and thermal properties of PLA films. In addition to the effectiveness of plasticizer, the stability of properties is an extremely important element for their applicability. Therefore, the influence of ageing must not be neglected. Consequently, the influence of accelerated ageing on mechanical, rheological, and thermal properties of PLA films plasticized with acetyl tributyl citrate (ATBC), polyethylene glycol (PEG), and nature-based dicarboxylic acid-based plasticizers (MC2178 and MC2192) were evaluated. The simulation of solar radiation at ground level that is defined in standard IEC 60068-2-5 evoked minimal changes in mechanical properties of PLA films plasticized with 15 wt. % of dicarboxylic acid-based plasticizer MC2192 and relatively low changes in rheological properties. Also, from the thermal analysis, no considerable changes were reported. In contrast, for PLA films plasticized with a dicarboxylic acid-based plasticizer of lower molecular weight (MC2178), some mechanical, rheological, and structural changes and migration tendencies were observed. Even greater changes in thermal and mechanical properties were achieved for PLA/ATBC. However, the highest changes were evaluated if the PEG plasticizer (15 wt. %) was incorporated into PLA film. The migration of PEG plasticizer could be ascribed as the major reason behind these changes.

Furthermore, the influence of plasticizer and accelerated ageing on biodegradation during the thermophilic composting (ISO 14855-1) of PLA films was evaluated. From the estimated results, the chemical composition and structure order (degree of crystallinity) could be highlighted as the most important factors. PEG plasticizer is a water-soluble hydrophilic plasticizer with polar groups (–OH). Consequently, intensive disintegration and biodegradation could be assumed. However, a low biodegradation rate was observed during the thermophilic composting. The used PEG 400 is characterized by a low molecular weight that evoked high structure order changes (high degree of crystallinity) in PLA films. Because the diffusion of water, as well as enzymatic degradation, takes place primarily in the amorphous phase, the low disintegration and microbial activity were evaluated. The aged PLA/PEG films evoked, when compared to as-produced films, a slightly higher biodegradation rate. The reason for this could be a high tendency of PEG plasticizer to migrate. The migration of plasticizer spurs a decrease in hydrogen bonding between PLA/PEG and ensures easy diffusion of water or oxygen. In contrast, the high molecular weight of dicarboxylic acid-based plasticizer MC2192 caused very low enhancement of the crystallinity degree of PLA films. Consequently, the higher content of amorphous parts and high molecular weight (lower interaction bonds with PLA) caused faster disintegration and biodegradation. The plasticizer MC2178, due to its lower molecular weight compared to MC2192, evoked higher structure order enhancement of as-produced PLA films. Therefore, the lower biodegradation rate of films was evaluated. The accelerated ageing of PLA/MC2178 films evoked some migration/releasing tendency of plasticizers that caused easier hydrolysis and increases in microbial activity (biodegradation rate). The influence of structure order could be further confirmed by the results of the biodegradation of as-produced PLA/ATBC films. The water-insoluble ATBC plasticizer evoked only low crystallinity degree enhancement of PLA films. Consequently, a faster biodegradation rate than for PLA films, with a higher structure order (as produced PLA/PEG, PLA/MC2178), was observed. After the accelerated ageing process, a significant increase in structure order and a minimal tendency to migration was observed. Therefore, a decrease of biodegradation rate was observed.

## Figures and Tables

**Table 1:** Properties of plasticizers.

Plasicizers	Molecular Mass (g∙mol^−1^)	Density (kg/m^3^)	Viscosity (mPas)	Sources
PEG 400	380–420	1.125 (at 20 °C)	30–45 (at 25 °C)	[23,24,25]
ATBC citroflex A4	402	1.048 (at 25 °C)	53.7 (at 25 °C)	[25,26,27]
MC 2178	1250	1.03–1.07 (at 20 °C)	650–750 (at 20 °C)	[28]
MC 2192	4236	1.04–1.10 (at 20 °C)	4000–6000 (at 20 °C)	[29]

**Table 2:** The individual proportions of total organic carbon of components.

Sample Designation	Proportions (%)
PLA	50
ATBC citroflex A1	59.7
PEG 400	60.0
MC2178	58.5
MC2192	58.5

**Table 3:** The evaluated results of Volume-Flow Rate (MVR) for as-produced and aged PLA films.

MVR (cm^3^/min^−1^)	Sample Designation				
	PLA	PLA/ ATBC	PLA/ PEG 400	PLA/ MC 2178	PLA/ MC 2192
Produced	13.9 ± 0.5	30.7 ± 0.1	3076 ± 216	23.9 ± 0.4	20.7 ± 0.1
Aged	14.5 ± 0.5	35.4 ± 0.1	1784 ± 43	32.0 ± 0.5	30.1 ± 0.4

**Table 4:** DSC data of neat and plasticized PLA films after production, accelerated ageing, and composting process.

Sample Designation		Exposition Time of Composting	T_g_ (°C)	T_cc_ (°C)	∆H_cc_ (J/g)	∆H_c_ (J/g)	T_c_ (°C)	T_m_ (°C)	∆H_m_ (J/g)	∆H (J/g)	X_C_ (%)
PLA	Produced	Initial	60.3	109.2	33.6	1.5	158.6	174.1	42.1	7.0	6.6
		14 days	59.8	-	-	1.4	161.9	175.4	51.3	49.9	47.0
		28 days	54.3	-	-	-	-	164.0	59.9	59.9	56.5
	Aged	Initial	60.1	106.3	33.2	2.3	159.4	174.8	41.7	5.4	5.1
		14 days	59.4	-	-	2.0	160.8	172.0	42.2	40.2	38.0
		28 days	54.5	-	-	-	-	165.5	59.7	59.7	56.3
PLA/ ATBC	Produced	Initial	41.7	86.4	23.8	6.4	146.6	171.3	42.7	12.5	13.9
		14 days	46.2	-	-	3.2	153.4	172.2	46.6	43.4	-
		28 days	-	-	-	-	-	170.4	49.4	49.4	-
	Aged	Initial	40.8	80.2	7.5	2.9	146.4	170.8	39.1	28.7	-
		14 days	44.9	-	-	2.3	160.8	172.1	41.7	39.4	-
		28 days	-	-	-	-	-	169.6	50.9	50.9	-
PLA/ PEG	Produced	Initial	37.6	84.1	13.4	1.7	-	167.9	41.3	26.26	29.5
		14 days	48.8	-	-	-	-	173.0	47.7	47.7	-
		28 days	-	-	-	-	-	172.0	48.0	48.0	-
	Aged	Initial	46.1	83.2	16.6	1.3	-	166.0	41.9	24.0	-
		14 days	-	93.9	6.9	-	-	168.8	50.9	44.0	-
		28 days	-	-	-	-	-	166.7	53.1	53.1	-
PLA/ MC 2178	Produced	Initial	40.1	89.5	22.8	5.0	150.7	172.5	44.3	20.9	18.2
		14 days	-	-	-	1.7	159.9	176.3	46.2	44.4	-
		28 days	-	-	-	-	-	168.8	55.5	55.4	-
	Aged	Initial	43.2	75.6	17.1	3.4	153.6	172.5	41.2	20.7	-
		14 days	-	-	-	-	-	172.0	46.8	46.8	-
		28 days	-	-	-	-	-	169.6	55.9	55.8	-
PLA/ MC2192	Produced	Initial	52.8	87.6	26.2	7.1	152.1	175.5	44.2	11.0	12.6
		14 days	-	-	-	-	-	163.4	59.2	59.2	-
		28 days	-	-	-	-	-	156.7	63.8	63.8	-
	Aged	Initial	51.5	80.6	26.4	5.9	151.1	174.4	45.5	13.2	-
		14 days	-	-	-	-	-	161.2	55.5	55.5	-
		28 days	-	-	-	-	-	156.8	60.6	60.6	-

**Table 5:** TGA data of neat and plasticized PLA films after production, accelerated ageing, and composting process.

Sample Designation	Exposition Time of Composting					
	Initial State		14 Days		28 Days	
	T_5_ (%)	T_50_ (%)	T_5_% (%)	T_50_ (%)	T_5_ (%)	T_50_ (%)
Produced PLA	313.4	349.9	310.9	342.5	293.9	330.6
Aged PLA	315.8	352.5	306.9	335.0	288.8	351.7
Produced PLA/ATBC	310.5	349.0	293.8	344.8	276.9	335.7
Aged PLA/ATBC	301.6	356.7	292.0	341.2	273.3	329.3
Produced PLA/PEG 400	259.4	321.7	250.4	301.6	244.1	282.9
Aged PLA/PEG 400	296.8	348.5	280.9	332.3	250.6	300.7
Produced PLA/MC2178	297.9	355.8	295.1	340.4	283.7	340.1
Aged PLA/MC2178	308.5	353.0	293.11	338.4	272.9	331.3
Produced PLA/MC2192	309.6	357.0	299.1	350.1	254.3	305.7
Aged PLA/MC2192	311.8	356.1	297.3	350.1	257.6	305.3

## Data Availability

The data presented in this study are available on request from the corresponding author.

## Supplementary Materials

The following supporting information can be downloaded at: https://www.mdpi.com/article/10.3390/polym15010140/s1, Figure S1: FTIR spectra of aged PLA films at initial state and after 14 days and 28 days of thermophilic composting; Figure S2: FTIR spectra of aged PLA films at initial state and after 14 days and 28 days of thermophilic composting that indicate hydroxyl bands; Figure S3: DSC curves of as produced neat and plasticized PLA films within 28 days of thermophilic composting; Figure S4: DSC curves of aged neat and plasticized PLA films within 28 days of thermophilic composting; Figure S5: TGA curves of as produced neat and plasticized PLA films within 28 days of thermophilic composting; Figure S6: TGA curves of aged neat and plasticized PLA films within 28 days of thermophilic composting.
