# Supplementary material for: The Influence of Plasticizers and Accelerated Ageing on Biodegradation of PLA under Controlled Composting Conditions
Source: Polymers (Basel). 2022 Dec 28;15(1):140. doi: 10.3390/polym15010140 (PMC9823598; doi:10.3390/polym15010140)
Supplement: Supplementary file 1 [file polymers-15-00140-s001.zip › polymers-2089085-supplementary.pdf]

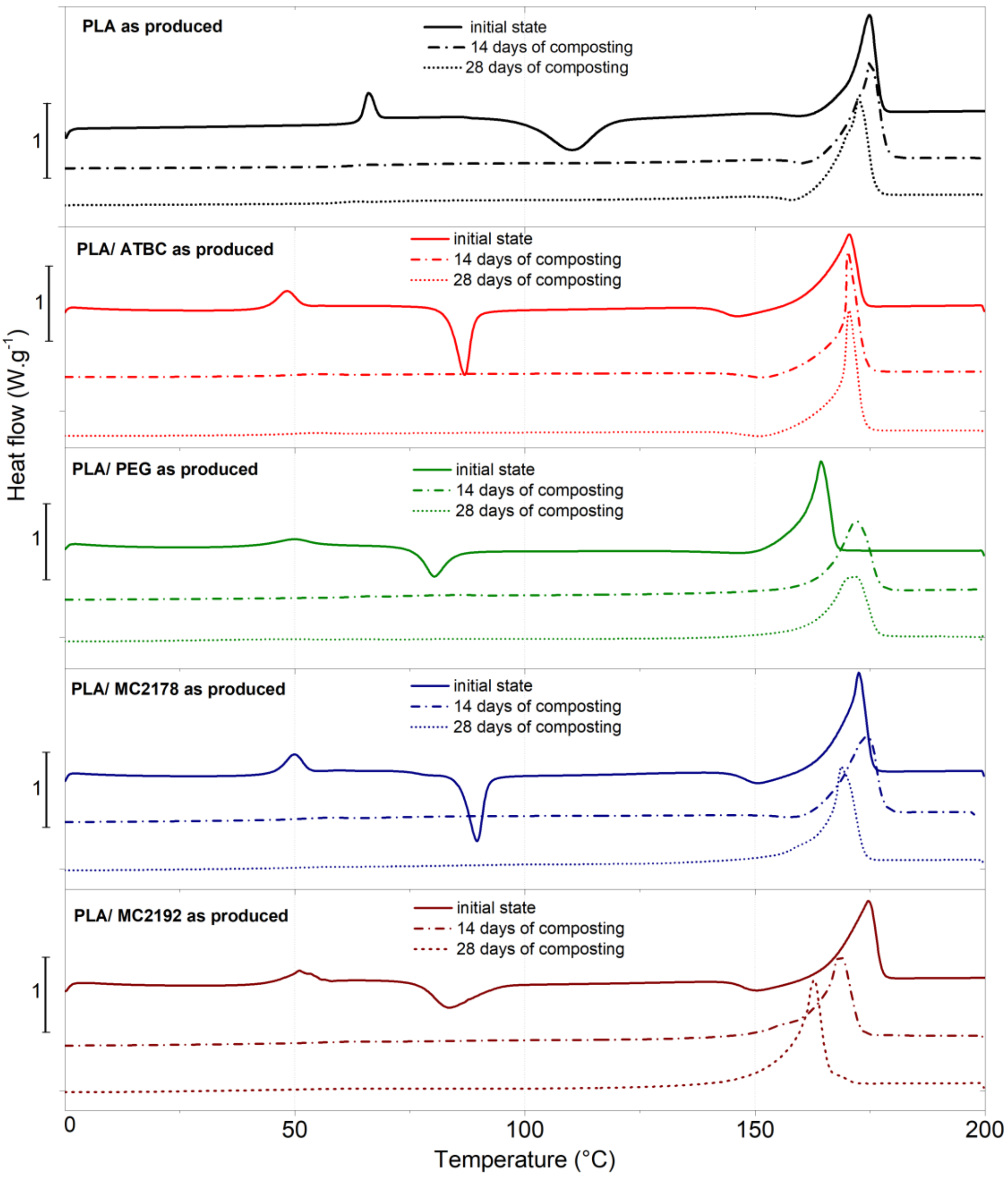

Figure S1: DSC curves of as produced neat and plasticized PLA films within 28 days of thermophilic composting.

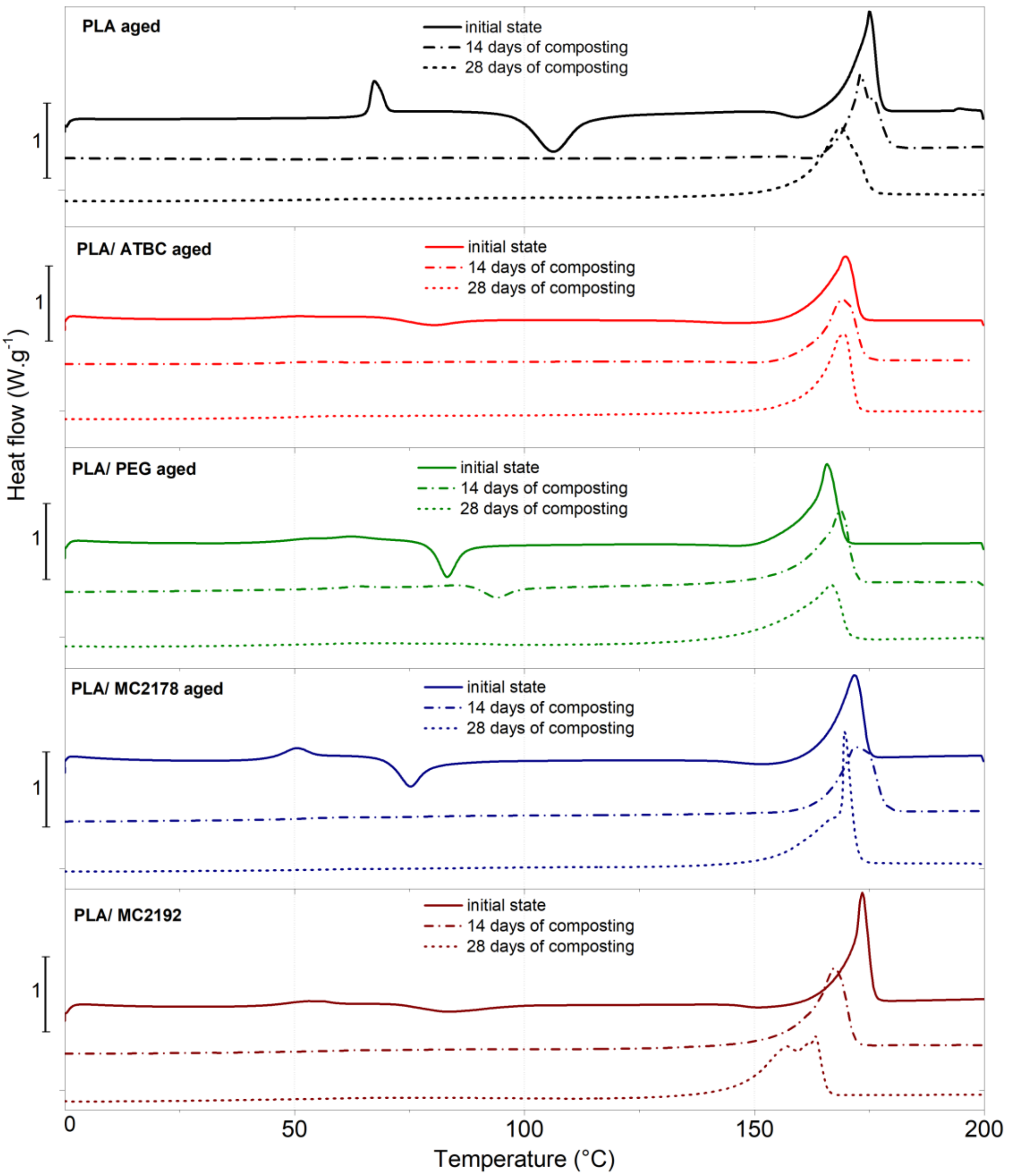

Figure S2: DSC curves of aged neat and plasticized PLA films within 28 days of thermophilic composting.

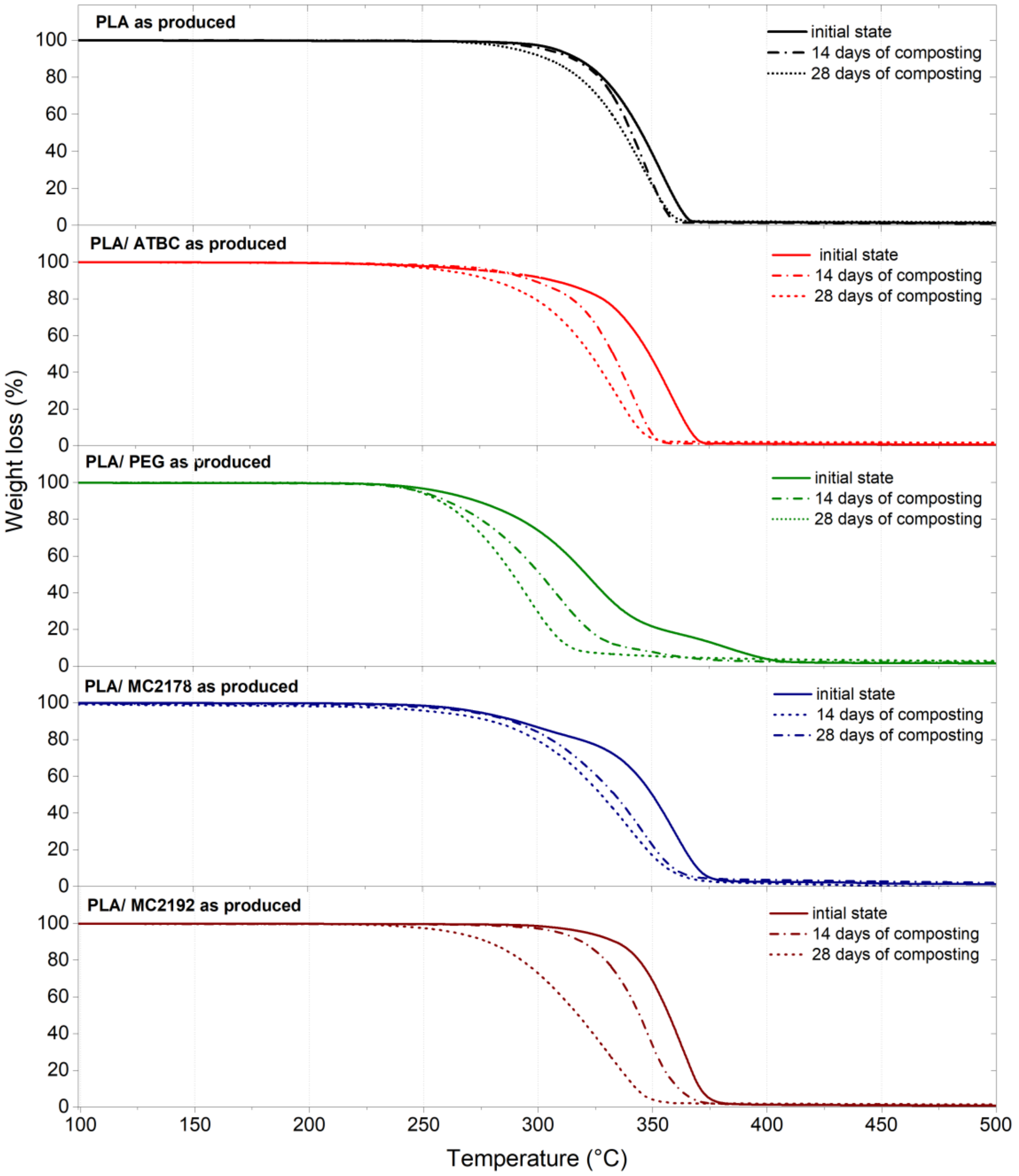

Figure S3: TGA curves of as produced neat and plasticized PLA films within 28 days of thermophilic composting.

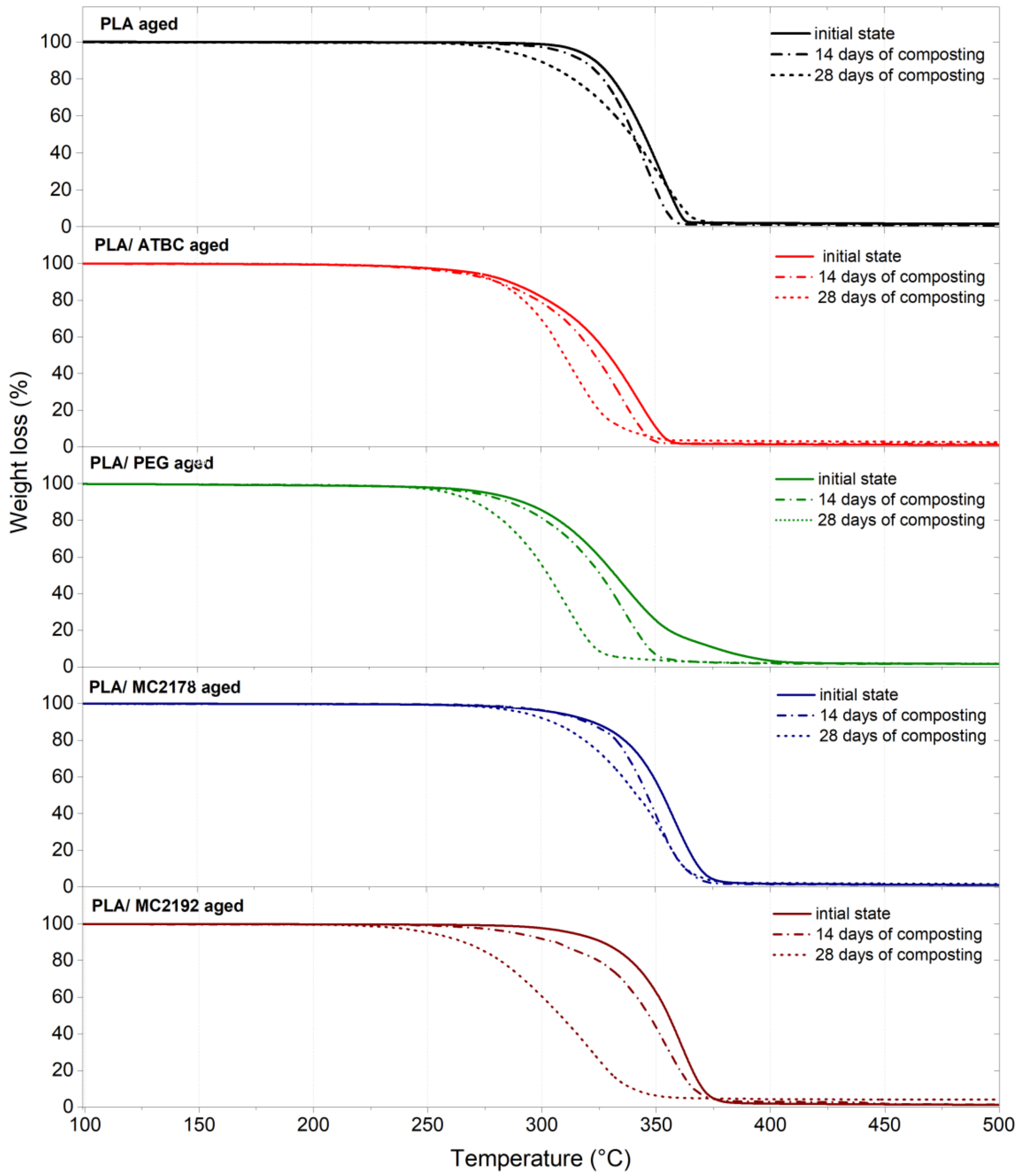

Figure S4: TGA curves of aged neat and plasticized PLA films within 28 days of thermophilic composting.

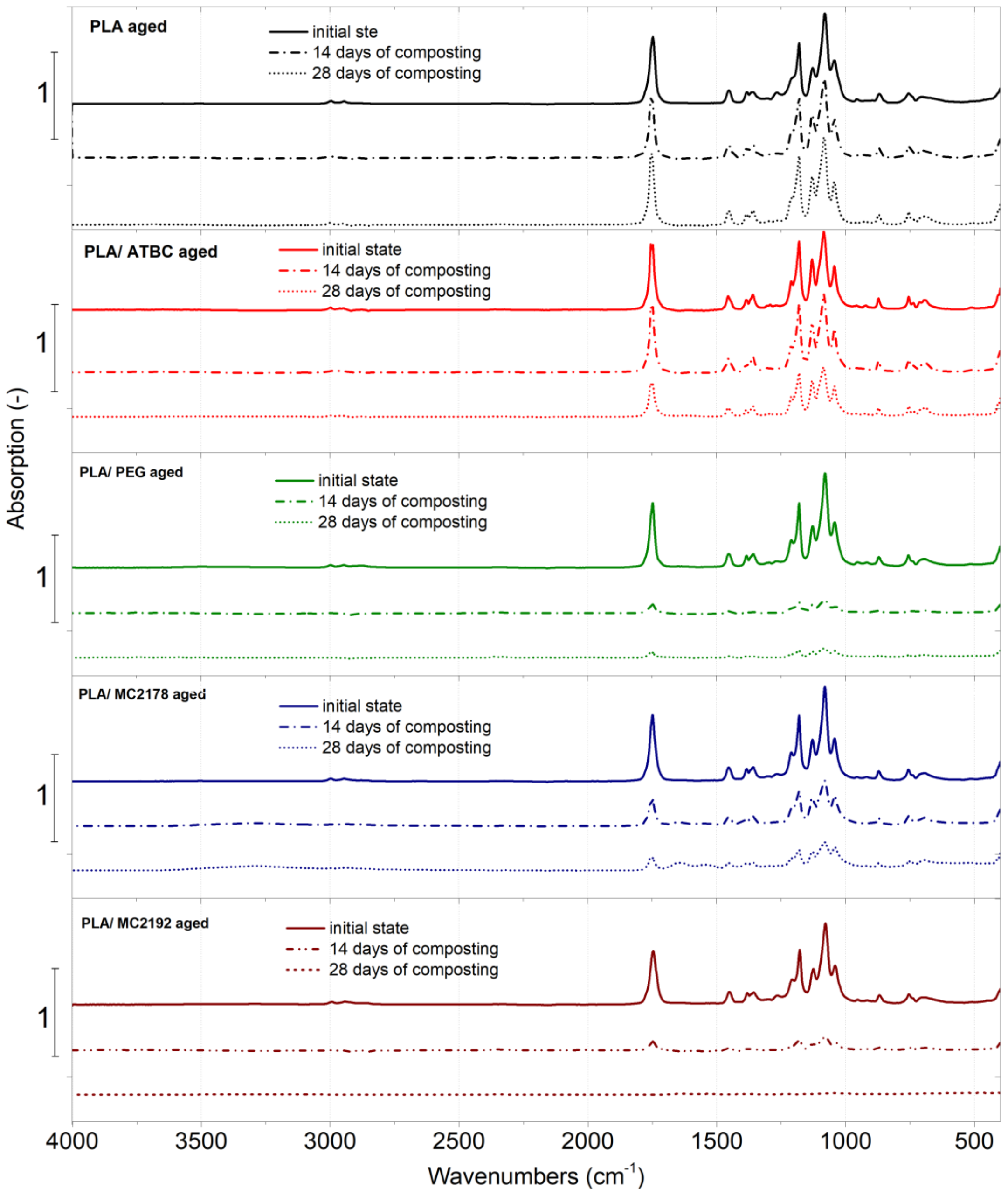

Figure S5: FTIR spectra of aged PLA films at initial state and after 14 days and 28 days of thermophilic composting.

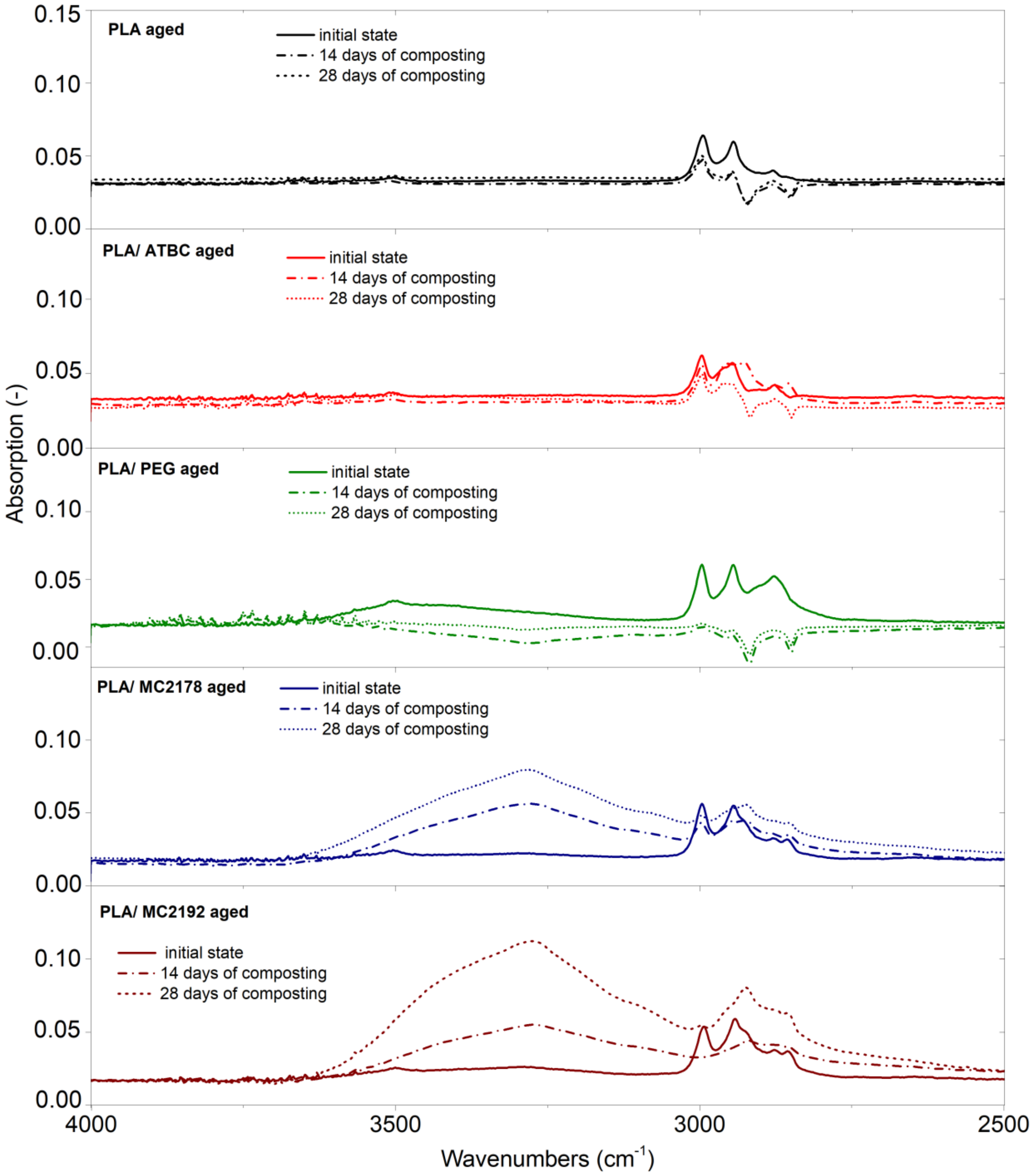

Figure S6: FTIR spectra of aged PLA films at initial state and after 14 days and 28 days of thermophilic composting that indicate hydroxyl bands.
